# Agro-Physiological and DNA Methylation Responses to Salinity Stress in Wheat (*Triticum aestivum* L.), *Aegilops cylindrica* Host, and Their Introgressed Lines

**DOI:** 10.3390/plants13192673

**Published:** 2024-09-24

**Authors:** Mohsen Hoseini, Ahmad Arzani, Ghodratollah Saeidi, Fabrizio Araniti

**Affiliations:** 1 Department of Agronomy and Plant Breeding, College of Agriculture, Isfahan University of Technology, Isfahan 84156-83111, Iran; moh.hoseiny@gmail.com (M.H.); gsaeidi@iut.ac.ir (G.S.); 2 Department of Agricultural and Environmental Sciences—Production, Landscape, Agroenergy, University of Milan, 20133 Milan, Italy

**Keywords:** wheat, salinity, DNA methylation, *HKT1;5*, *NHX1*, *SOS1*

## Abstract

Bottlenecks, including limited genetic variation and the ongoing loss of genetic diversity, have hindered the development of modern wheat cultivars., making it crucial to use genetic diversity from wild relatives to improve wheat’s adaptation to abiotic stress, such as salinity. This study assessed the phenotypic and epigenetic variation of introgressed wheat lines (BC_4_F_2_) derived from hybridizing two wheat cultivars with *Aegilops cylindrica* (AC). This study assessed the phenotypic and epigenetic variation of 156 introgressed wheat lines (BC_4_F_2_) derived from hybridization between wheat cultivars “Chinese Spring” (CS) and “Roshan” (R) and *Aegilops cylindrica* (AC). These lines and their recurrent parents (total of 158) were evaluated under normal and saline field conditions for the agronomic traits and stress tolerance indices. The data were used to select the most tolerant and most sensitive lines. Then, the selected BC_4_F_2_ lines and their parents (AC, CS, and R) were subjected to physiological, DNA cytosine methylation, and expression analysis of *HKT1;5*, *NHX1*, and *SOS1* genes under control and salt stress conditions. Agro-physiological, epigenetic, and gene expression analyses showed the significant effects of salt stress and genetic background, as well as the differential response of the BC_4_F_2_ lines to salt stress. The variations in leaf and root K, Na, and K/Na ratios, and leaf Chla, Chlb, Car, and MDA levels, unlike DPPH radical scavenging levels, between salt-tolerant and salt-sensitive BC_4_F_2_ lines under saline conditions indicated a substantial distinction in salinity tolerance responses. RT-qPCR indicated higher expression levels of *NHX1* and *SOS1* genes in the leaf and root tissues of tolerant lines than those of sensitive lines. Global leaf and root DNA methylation analysis revealed the significant effects of salinity on the methylation modifications and confirmed the successful introgression of the salt-tolerance epigenome from *Ae. cylindrica* into wheat. Exploiting the genetic diversity of wild wheat relatives is a crucial goal for increasing genetic and epigenetic variation to enhance plant adaptation to salt stress.

## 1. Introduction

Increasing wheat productivity is crucial due to the rising food demand driven by a growing population. However, efforts to enhance photosynthesis and crop yields have been hindered by the impacts of climate change and the erosion of plant genetic resources [1]. The salinity of agricultural land is expected to increase due to insufficient drainage, excessive use of fertilizers, global warming, rising water levels, lack of rainfall, and irrigation with saline water [2,3]. Consequently, salinity has become a significant abiotic stressor in modern agriculture, presenting a major limitation to production as the need arises to utilize new resources like saline soil and water [4].

While increasing crop yields in areas with unfavorable conditions through agronomic solutions is often unsustainable, the genetic improvement of crop tolerance to abiotic stressors offers a cost-effective and sustainable approach to stabilizing and increasing productivity [1,5]. Hexaploid wheat (*Triticum aestivum* L.), such as bread wheat (ABD genomes), shows less sensitivity to salt compared with durum wheat (AB genomes) [6]. The near-complete homology of wheat salt tolerance genes such as *P5CS* [7] and *HKT* [8] with those in *Aegilops tauschii*, coupled with the dominant presence of these genes on the wheat D genome, suggests that salt tolerance in bread wheat is linked to its donor origin in *Ae. tauschii. Ae. cylindrica*, another species with the D genome, is considered a significant potential source of salinity tolerance among the 22 *Aegilops* species [1]. This species (*Ae. cylindrica*), a typical salt-excluding halophyte, exhibits excellent salt tolerance [9] and shares a common D genome with common wheat (*T. aestivum* L.). Therefore, wild ancestors of wheat are crucial for the continuous improvement of bread wheat [10].

Due to the polygenic nature of salinity tolerance and the effect of this stress on plants through three phases of initial osmotic-stress phase, followed by ionic toxicity from sodium (Na^+^) and chloride (Cl^−^) accumulation in the cell cytosol and culminating in oxidative stress [1,9,11,12,13], there are at least three crucial mechanisms that enhance plant tolerance to salinity. These mechanisms are protection against salt-induced osmotic stress, the regulation of Na^+^ absorption and transport in roots and shoots, and Na^+^ exclusion from the cytosol, which promotes tissue tolerance [14]. These stress effects induce biochemical changes at the genomic level and physiological responses in plant tissues. Increasing the capacity for osmotic adjustment under stress conditions by accumulating compatible solutes such as proline and soluble sugars may improve crop salt tolerance [9,15]. In addition, reactive oxygen species (ROS) are produced in saline conditions, leading to an increase in malondialdehyde (MDA) by stimulating lipid peroxidation [16].

In addition to managing Na^+^ ion cytotoxicity, another important mechanism by which plants tolerate salinity stress is by maintaining intracellular Na^+^ and K^+^ homeostasis in photosynthetically active tissues [17,18,19]. Na^+^ toxicity is largely assumed to result from disrupting cytoplasmic enzymatic activities, as Na^+^ occupies K^+^ binding sites in key enzymes. Thus, disturbance of this homeostasis leads to the inhibition of photosynthesis, cell division, growth, and development [20,21]. For these reasons, thigh ratios of K^+^/Na^+^ are recognized as a decisive physiological mechanism for overall salt tolerance in many crop plants [19,22,23]. In this mechanism, unlike K^+^, Na^+^ does not accumulate in leaves to toxic levels by discriminating exclusion [16,24]. Genetic variation is significant in this mechanism of discriminately excluding Na^+^ [25].

The exchangers of plasma membrane (PM)-localized salt-overly sensitive 1 (*SOS1*), Na^+^/H^+^ antiporter, tonoplast-localized Na^+^/H^+^ antiporter (*NHX1*), and the high-affinity potassium transporters (*HKT*) proteins [11,26] together with osmoprotectant pyrroline-5-carboxylate synthetase (*P5CS*) [7] are likely important factors contributing to salt tolerance in this species. Thus, improving crop tolerance to salt stress conditions necessitates a genetic and epigenetic dissection of the multifaceted mechanisms involved in plant salt tolerance.

Epigenetic modulation provides plants with the ability to adapt to challenging environmental conditions. Epigenetic modifications include DNA methylation, histone modifications, and non-coding RNAs [27]. DNA methylation plays an important role in gene expression regulation in the response of plants to abiotic stresses [27,28]. Changes in the DNA methylation patterns during a lifetime provide an adaptive ability to environmental change for the plants [29,30,31]. Epigenetic variations in DNA, assessed through methylation modifications during a plant’s life under different conditions, have become valuable markers for selecting genotypes better adapted to stress. More recently, there are reports of DNA methylation alterations in cereal plants exposed to abiotic stress conditions, including salinity [32,33], drought [34,35], and temperature stress [30].

Building on our previous work in screening a hyper-salt tolerant genotype of *Ae. cylindrica* [16,24] and hybridizing it with two wheat cultivars, along with cytological [36] and molecular evaluations of the synthesized amphidiploids [7,11]. This study aims to investigate the following: (i) screen salinity-tolerant BC_4_F_2_ lines from various genetic backgrounds; (ii) assess their agronomic and physiological responses to salinity stress; (iii) investigate epigenetic changes caused by salinity; (iv) and analyze the expression of key salinity tolerance genes.

## 2. Materials and Methods

### 2.1. Plant Materials

F1-amphidiploid plants derived from “Chinese Spring” and “Roshan” (hereafter called CS and R, respectively) wheat cultivars × *Ae. cylindrica* (hereafter called Ac) were backcrossed to their wheat parent to generate BC_4_F_1_ lines. These BC_4_F_1_ lines were then selfed to increase the seeds for two groups of BC_4_F_2_ lines. One hundred fifty-eight genotypes were used in this study, including their parents (CS, R, and salinity-tolerant Ac genotype). These include 72 BC_4_F_2_ lines derived from CS × Ac and their replicate parents (CS), as well as 84 BC_4_F_2_ lines derived from R × Ac along with their replicate parents (R).

### 2.2. Field Experiment

Field experiments were conducted over two years (2021–2022 and 2022–2023) under normal and saline environments at the research farm of Isfahan University of Technology, located at Lavark, Iran (32°32′ N and 51°23′ E; 1630 m asl). A randomized complete block design was used for each experimental environment. Management of wheat plants was similarly carried out following standard grower practices, including fertilizer application and weed control. Irrigation water was delivered from a pumping station to the plots via polyethylene pipes equipped with water flow meters. Salt treatment was initiated at the 4-tiller stage with initially diluted NaCl irrigation water (125 mM), which was subsequently increased to a concentration of 250 mM NaCl. The top 60 cm layer of the field soil consisted of clay loam soil with a pH of 7.5 and an average EC of 4.2 dS m^−1^ at the time of planting. However, by the time of harvest, the experimental plots treated with salt had an average soil EC of 13.3 dS m^−1^.

### 2.3. Agronomic Traits

Agronomic traits include plant height (PH), spike length (SL), number of spikes per plant (SpP), number of grains per spike (GpS), 100 grain weight (GW), and grain yield per plant (yield) were evaluated under the two normal and salt stress conditions.

### 2.4. Salinity Tolerance Indices

The grain yield data obtained from normal (0 mM NaCl) and saline field (250 mM NaCl) conditions were used to calculate the indices, according to the equations below. The stress tolerance index (STI) [37], tolerance index (TOL) [38], stress susceptibility index (SSI) [39], yield stability index (YSI) [40], yield index (YI) [41], geometric mean productivity (GMP) [39], and mean productivity (MP) [38], harmonic mean (HM) [42], and Mean Relative Performance (MRP) [43].

1. Stress tolerance index:

$$STI=\frac{\left(Yn\times Ys\right)}{{\bar{Yn}}^{2}}$$

2. Tolerance index:

TOL=Yn−Ys

3. Stress susceptibility index:

$$SSI=\frac{\left[1-\left(\frac{Ys}{Yn}\right)\right]}{SI}$$

“SI” stands for “stress intensity” and is calculated using the following formula:


$$SI=1-\frac{\bar{Ys}}{\bar{Yn}}$$

4. Yield stability index:

$$YSI=\frac{Ys}{Yn}$$

5. Yield index:

$$YI=\frac{Ys}{\bar{Yn}}$$

6. Geometric mean productivity:

$$GMP=\sqrt{\left(Yn\times Ys\right)}$$

7. Mean productivity:

$$MP=\frac{\left(Yn+Ys\right)}{2}$$

8. Harmonic mean:

$$HM=\frac{2(Yn\times Ys)}{Yn+Ys}$$

9. Mean Relative Performance:

$$MRP=\frac{Yc}{\bar{Yc}}+\frac{Ys}{\bar{Ys}}$$

$\bar{Yn}$ and $\bar{Ys}$ denote the average yield of all genotypes with the same genetic background (CS or R) under normal and salinity stress conditions.

A new index named “Yield Loss Index” (YLI) is proposed in this paper as a measure of tolerance. YLI for each genotype is calculated by dividing the yield loss of each genotype due to the stress environment (Yn−Ys) by Yn.

10. Yield loss index

$$YLI=\frac{Yn-Ys}{Yn}$$

### 2.5. Physiological Traits

The following physiological traits were evaluated in the leaf and root tissues in this study.

### 2.6. Proline Content

Free proline was extracted from fresh leaves and roots, and absorbance was measured after derivatization with acid ninhydrin, following the method described by Bates et al. [44]. Briefly, fresh leaves (0.5 g) were frozen in liquid nitrogen, ground, homogenized in 10 mL of sulfosalicylic acid (3% w v^−1^), and centrifuged for 5 min at 8600× *g*. Then, 2 mL of supernatant was mixed with 2 mL of ninhydrin reagent and 2 mL of acetic acid. After heating for an hour at 100 °C, 4 mL of toluene was added. Absorbance was measured at 520 nm using a spectrophotometer (Shimadzu UV-Vis 1201), and proline concentration was determined using a standard curve based on the following formula:

$$\mathrm{Proline}\;(µM/g\;\mathrm{FW})=\frac{(µg\;Proline/mL\times mL\;Toluene)\times 5}{\left(115.5\;µg/µM\times g\;Sample\right)}$$

### 2.7. Total Soluble Sugars (TSS)

To determine the leaf and root soluble sugar content, we used the method described by Irigoyen et al. [45]. Initially, an alcoholic extract was obtained using 0.5 g of plant tissue and 5 mL ethanol, which was then centrifuged at 2000× *g* for 10 min. The soluble sugars were quantified using anthrone and sulfuric acid (72%) and a standard curve. Absorbance of the samples was measured after 10 min in a boiling water bath, followed by cooling to room temperature, and ultimately reading at 625 nm using a spectrophotometer. Anthrone reagent was used, and distilled water was the blank.

### 2.8. Malondialdehyde (MDA)

Malondialdehyde (MDA), as an indicator of lipid peroxidation in leaf and root tissues, was quantified in leaf homogenates using the thiobarbituric acid (TCA) test [46]. Briefly, 300 mg of fresh leaf were frozen with liquid nitrogen, ground, and homogenized in 5 mL of a 0.1% TCA. After centrifugation of the homogeneous mixture at 12,000× *g* for 10 min at 4 °C, 0.5 mL of the supernatant was mixed in 2 mL of 20% TCA containing 0.5% thiobarbituric acid (TBA). The samples were then heated for 30 min at 95 °C in a water bath and immediately cooled on ice. Finally, the absorbance of the supernatant obtained from centrifugation at 10,000× *g* for 10 min was measured at wavelengths 532 and 600 nm. The absorbance at 600 nm was subtracted to correct for non-specific turbidity. MDA content was calculated using the extinction coefficient of 155 mM^−1^ cm^−1^ according to the following formula:

$$MDA\left(nM\right)=\frac{{\Delta A}_{\left(532–600\right)}}{{1.56\times 10}^{5}}$$

### 2.9. DPPH Radical-Scavenging Activity

The procedure described by Kiani et al. [9] was used to determine the DPPH radical scavenging activity of the leaf and root samples as an indicator of antioxidant activities. Briefly, 0.1 mL of the sample of fresh leaf extract was blended at selected concentrations (50, 100, and 300 ppm). The initial and final absorbance values of DPPH in the BHT standard were within the accurate range of spectrophotometry [47]. For optimal results, 5 mL of 0.1 mM methanol DPPH solution was selected as the sample volume. Next, the sample vials were shaken vigorously, and the absorbance was measured at 517 nm (AA) after incubating for 30 min at room temperature in the dark. The absorbance of blank reagent (AB), which is the DPPH-methanol solution containing 80% methanol, was used to correct the AA absorption and served as the negative control. The positive control in the experiment was a synthetic antioxidant reagent, butylated hydroxytoluene (BHT). The IC50 value (µg mL^−1^), the concentration in µM at which 50% of DPPH absorption is inhibited, was determined through linear regression analysis.

### 2.10. Chlorophyll and Carotenoid Content

The chlorophyll (a + b) and carotenoid (c) content were determined through the 80% acetone extract obtained from fresh leaf tissue using the spectrophotometric method and the equations introduced by Lichtenthaler and Buschmann [48]. About 500 mg of the middle part of the flag leaf was homogenized in 5 mL of 80% acetone and stored in the dark for 5 min. After filtering the samples, the absorbance at 645, 663, and 470 nm was measured with 80% acetone as the blank solution. The concentrations of chlorophyll a (Chla), chlorophyll b (Chlb), total chlorophyll (Total Chl), and carotenoids (Car) were quantified in mg × g^−1^ FW using the given formulas:

$$Chlorophyll\;a=\frac{\left[\left(12.7\times A_{663}\right)-(2.69\times A_{645})\right]}{1000\times W}$$

$$Chlorophyll\;b=\frac{\left[\left(22.9\times A_{645}\right)-(4.69\times A_{663})\right]}{1000\times W}$$

Total chlorophyll=Chlorophyll a+Chlorophyll b

$$carotenoids=\frac{\left[\left(1000\times A_{470}\right)-\left(1.82\times Chl\;a\right)-(85.02\times Chl\;b)\right]}{198}$$

### 2.11. Leaf and Root Na and K Concentrations

Leaf and root samples were dried at 80 °C for 48 h until they reached a constant weight. Next, 0.2 g of dried samples were incinerated in a muffle furnace at 550 °C for 4 h to determine leaf and root Na and K concentrations. To extract mineral ions, each of the obtained ash samples was dissolved in 10 mL of 2 N HCl, and the final volume was made to 100 mL. Na and K concentrations of the solutions were determined using a standard curve [49] by flame photometry PFP7 (Jenway, England). The K/Na ratio was accordingly calculated.

### 2.12. Epigenetic and Gene Expression Analyses

Sixteen genotypes were selected and used for the epigenetic and RT-qPCR experiments. These include 3 tolerant and 3 sensitive BC_4_F_2_ lines, the donor parent (*Ae. cylindrica*), and the recurrent parent, in each of the two backgrounds (CS and R). The seeds were planted in pots with a height of 30 cm and a diameter of 20 cm, containing soil and sand mixtures in a 3:1 ratio. After germination at 4 °C, the pots were transferred to a greenhouse with a relative humidity of 60–65%, an average daytime temperature of 26 ± 4 °C, a nighttime temperature of 18 ± 4 °C, and a photoperiod of 12 h with a photosynthetic photon flux density (PPFD) of approximately 380 μmol m^−2^ s^−1^. At first, all the pots were irrigated with drinking water (EC = 1.3 dS m^−1^). The salinity treatment was initiated at the second leaf stage with half-strength salt, followed by gradually adding NaCl until the concentration reached 250 mM, which was maintained for 72 h. A 3 × 8 factorial experiment in a completely randomized design layout replicated three times was used for each genetic background. The first factor comprised two NaCl treatments (0 and 250 mM NaCl), while the second and third factors comprised 8 genotypes and two types of tissues (leaf and root), respectively. Two additional technical replicates were used for gene expression analysis. Leaf and root samples from the experimental units were immediately liquid nitrogen snap-frozen and stored at −80 °C until use.

### 2.13. DNA Methylation Analysis

The leaf and root genomic DNA were extracted using the CTAB method [50], and the air-dried pellet was re-suspended in 50 µL TE buffer (10 mM Tris, pH = 8, 1 mM EDTA). Twenty microliters of DNA solution between 200 and 1700 ng/µL were transferred to an HPLC vial. Sixty microliters of concentrated formic acid were added to the vial and vortexed. DNA hydrolysis was performed by heating the HPLC vial in a drying oven at 130 °C for 3 h. After the vials had cooled, 100 µL of water was added to each vial and vortexed to mix. One microliter of the resulting sample hydrolysate solution was injected into a Vanquish core HPLC system (Thermo Fisher Scientific Inc., Waltham, MA, USA) with a UV-VIS diode array detector. The HPLC column was a Hypercarb (Thermo Fisher Scientific Inc., Waltham, MA, USA), with dimensions of 50 mm × 2.1 mm i.d., a 3 µm particle size, and a temperature of 40 °C. The autosampler flush solvent and the flow rate were 10% methanol (*v*/*v*) and 0.2 mL/min, respectively. The wavelength of UV detection was 295 nm. Mobile phase A and phase B consisted of water with 0.1% formic acid (*v*/*v*) and acetonitrile with 0.1% formic acid (*v*/*v*), respectively. The elution gradient followed the sequence below: 0–14 min, 0% B to 35% B; 14–14.1 min, 35% B to 0% B; 14.1–20 min, 0% B. The runtime was 20 min. The HPLC was calibrated using the equimolar solution of cytosine and 5-mC containing 50 µM in 10% methanol (*v*/*v*). To obtain the relative content of 5-mC in the sample solutions, we calculated the peak area ratio of 5-mC–cytosine.

### 2.14. RNA Extraction and cDNA Synthesis

Total RNA was isolated from powdered leaf and root samples (100 mg) in liquid nitrogen by the CTAB method [51] with minor modifications. Briefly, after incubating the homogenized samples with extraction buffer (2% CTAB, 2% PVP-40, 20 mM EDTA pH 8.0, 1.4 M NaCl, 0.1 M Tris–HCl pH 8.0, and 10% of β-mercaptoethanol added immediately before use) at 65 °C for 10 min, 8.00 mL of chloroform was added to the tube before its vigorous inversion and centrifugation at 10,000× *g* for 10 min at 4 °C. The supernatant phase was transferred to a new tube containing an equal volume of chloroform and phenol (1:1) and centrifuged at 11,000× *g* for 10 min at 4 °C. Then, similar to the previous step, the supernatant was mixed with an equal volume of chloroform–isoamyl alcohol (24:1 *v*/*v*) and centrifuged. After treating total RNA with DNase and measuring the purity by ND-1000 NanoDrop (Thermo Scientific, Wilmington, DE, USA), Synthesis of cDNA from 1 µg RNA was carried out using Suprime -Script RT premix (GeNet Bio Inc., Daejeon, Republic of Korea) according to the manufacturer’s instructions.

### 2.15. Quantitative Real-Time PCR

The expression profiles of *HKT1;5*, *NHX1*, and *SOS1* genes were analyzed with their specific primer pairs using the quantitative real-time PCR (qRT-PCR) technique. The RT-qPCR was performed in 20 µL of a reaction mixture containing 1 µL of diluted cDNA, 5 µL of 2 × Fast SYBRGreen PCR Master Mix (Applied Biosystems), 0.2 µL of 1 µM each of the reverse and forward primers, and 13.6 µL of RNAase-free water. All amplification reactions were performed in duplicate in optical 96-well plates (Applied Biosystems, Foster City, CA, USA) with reverse transcriptase negative controls to confirm the absence of genomic DNA contamination according to the following cycling protocol: 94 °C for 4 min, 42 cycles of 95 °C for 30 s, 60 °C for 35 s, 72 °C for 1.5 min, and 72 °C for 10 min. The housekeeping gene β-actin (ACTB) was used as a reference gene to normalize the expression of the studied genes. Real-time PCR data of the target mRNA were analyzed using the 2^−ΔΔCT^ method [52]. The relative expression levels of the target samples were calculated using the ΔΔCT method and expressed relative to the values in the normal tissues after normalization.

### 2.16. Statistical Analyses

The resulting data from each group of backcross lines grown each year were assessed for normality and homogeneity of variance before analyzing variance (ANOVA) using SAS version 9.4M7 (SAS Institute Inc., Cary, NC, USA). Subsequently, a combined analysis of variance was carried out using SAS PROC.MIXED. Fisher’s protected least significant difference (LSD_0.05_) test was used to detect significant differences among the means of the variables. Correlation was performed to delineate the relationships among the traits using the “ggplot2” package of R software version 4.4.0. For each genetic background, principal component analysis (PCA) was carried out for tolerance indices obtained from grain yield under stress (250 mM NaCl) and normal conditions. The first two PCS (PC1 and PC2) were used to construct the genotype-by-yield biplot using “Factoextra” packages of R software. To achieve a more stable clustering of genotypes based on salinity tolerance and to determine the selection range on the biplot, we first utilized principal component analysis (PCA) for genotype clustering (hierarchical principal component clustering (HCPC)). The resulting cluster data were then applied to the biplot drawing. The Venn diagram was then used to select the most tolerant and sensitive lines with common clusters over the two years based on the HCPCs. A linear regression analysis examined the relationship between crucial physiological traits related to salt stress resilience and grain yield.

## 3. Results

### 3.1. Grain Yield and Related Traits

The ANOVA results indicated significant effects (*p* ≤ 0.01) of salinity stress on grain weight, grain per spike, spikes per plant, spike length, grain yield, and plant height in CS and R-derived BC_4_F_2_ lines (Table 1). Significant differences between the two growing years were observed for grain yield and yield-related traits, except for spike length in the CS background. In contrast, in the R background, only grain weight and plant height showed significant differences between the two years. Furthermore, the results indicated that the genotypes differ significantly. The genotype-by-stress interaction was also significant for grain yield and related traits (except spike length). Although the genotype-by-year interaction was significant for the number of spikes per plant and plant height in the CS background, none of the studied agronomic traits were affected in the R background (Table 1).

Table 2 presents the Pearson correlation coefficients for agronomic traits of the BC_4_F_2_ lines in normal and saline field environments. SpP was the only yield component that highly and consistently correlated with yield in both introgressed line groups and under normal and stressful conditions. PH, an important plant growth trait, had a similarly strong and consistent relationship with yield. In the normal environment, SpP negatively correlated GW, GpS, and SL in both genetic background lines. Under the salinity environment, while the yield of the CS background and all studied agronomic traits showed a positive correlation, the GW with yield and two other yield components exhibited negative correlations in the R background. Additionally, SpP with GpS in both genetic background lines and GW, SL, and PH in the CS background correlated negatively. All traits in the CS background lines correlated positively, while the GW negatively correlated with yield, GpS, SL, and PH in the R lines.

When both BC_4_F_2_ line groups were exposed to 250 mM NaCl, the strongest effect of salinity stress appeared in the yield reduction. However, the weakest deleterious effects of salinity were observed for SL in the CS background and for both SL and GW in the R background. The overall decrease in grain yield was 49.5% in the CS lines and 45.9% in the R lines. SL decreased by 7.2% in the CS lines and 9% in the R lines, while grain weight decreased by 26% in the CS lines and 9% in the R lines.

**Figure 1:**
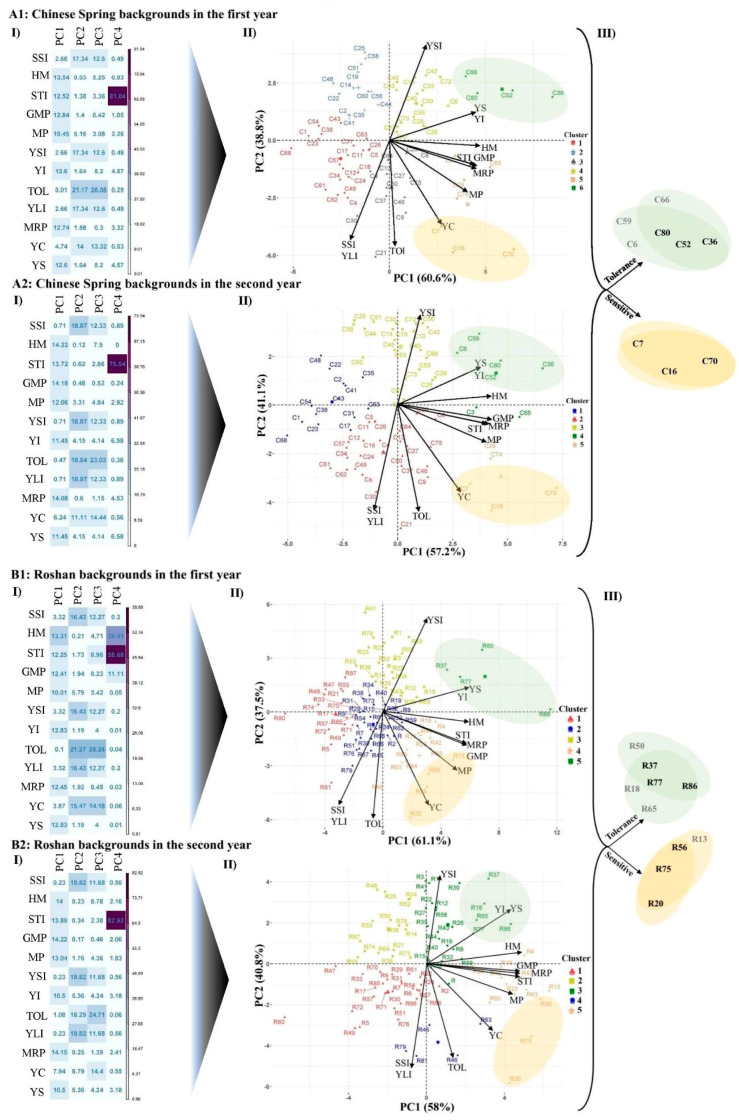
Hierarchical clustering based on principal component analysis (PCA) and Venn diagram using tolerance indices for selecting wheat genotypes tolerant to salinity. (I): contributions of variables (different indices) to PCs. (II): cluster plot of genotypes distributed in the PCA. (III): the Venn diagram to select the most tolerant and sensitive genotypes over the two years.

Table 3 shows the grain yield and tolerance indices of the tolerant and sensitive BC_4_F_2_ lines selected based on the weighted mean of yield, Tol, YLI, and SSI. Among the CS background lines, C16, C21, and C30 were identified as the most tolerant introgressed lines. Similarly, R20, R79, and R81 were identified as the most tolerant among the R background lines. The biplot diagrams, based on data from 73 CS lines and 85 R lines, respectively, illustrate the relationships between variables and between the lines and the variables (Figure 1). The backcross lines with the highest stable yield were identified using the stability index (YSI). The most stable high-yielding lines were C25, C42, and C72 from the CS background and R1, R39, and R41 from the R background.

The PC analysis identified the primary contributors to the total variation in yield and yield-based tolerance indices (Figure 1). Under salinity conditions, 60.6% of the total variation in CS lines during the first season and 57.2% during the second season were explained by PC1. At the same time, PC2 explained a smaller portion of the variation along the same lines, accounting for 38.8% and 41.1% in the first and second seasons, respectively. For the R lines under the same environmental conditions, PC1 explained 61.1% of the total variation in the first and 58% in the second seasons. PC2 explained 37.5% and 40.8% of the variation in the first and second seasons, respectively. PC1 was termed “yield potential” due to its strong relationships with YI, HM, STI, GMP, MRP, and MP indices. At the same time, PC2 was named “stress tolerance,” given its high associations with TOL, SSI, and YLI and its negative correlation with YSI. The most salinity-tolerant lines were located in the high PC1 and high PC2 areas of the biplot. In contrast, most salinity-sensitive lines were located in the lower right quadrant of the biplot (see Figure 1). Accordingly, based on their similarity in the biplot locations derived from grain yield and tolerance indices over two years, the C7, C16, and C70 lines from the CS group and R20, R56, and R75 lines from the R group were selected as the most sensitive lines. Similarly, the C36, C52, and C80 lines from the CS group and the R37, R77, and R86 lines from the R group were selected as the most tolerant lines (Figure 1). Table 3 presents the overall means (over two years) of grain yield of the superior introgressed wheat lines derived from wheat × *Ae. cylindrica* (“CS” and “R” wheat cultivars as the two recurrent parents) under normal (Yn) and salinity stress (Ys) conditions, as well as selection indices.

### 3.2. Physiological Responses of Introgressed Lines to Salinity

#### 3.2.1. Proline Content in Leaf and Root

The ANOVA results presented in Table 4 showed significant effects of salt stress, genotype, and their interaction on leaf and root proline content in both studied genetic backgrounds. Exposure to 250 mM NaCl led to a substantial increase in proline content in the leaves BC_4_F_2_ lines of two wheat backgrounds and their donor parent (Ac) (Table 5). The highest increase in this trait was observed in Ac, while the lowest was found in the R background lines. In contrast, the roots of the lines showed a weaker response to proline accumulation than the leaves when plants were exposed to salinity. Under normal conditions, the Ac and wheat lines’ leaf and root proline content remained constant, with minor alterations. The highest proline accumulation in leaf and root tissues occurred in the donor parent (Ac) under normal and salt stress conditions. They were significantly higher than that observed in the wheat parents. Although the leaf phenotype of all selected backcross plants (except for the tolerant line C70) under stress conditions was higher than that of their female parents (CS or R), this trend was not consistent for root proline content. Notably, the leaf and root proline content of all selected lines was lower than that of their respective recurrent parent (CS or R) under control conditions (Supplementary Figure S1).

#### 3.2.2. Total Soluble Sugar (TSS) in Leaf and Root

**Figure 2:**
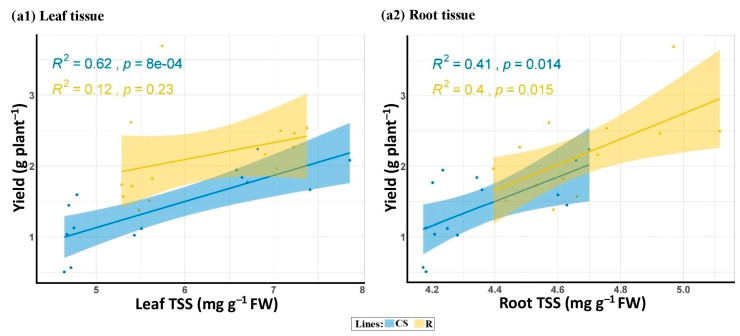
Linear relationship between grain yield (g/plant) and total soluble content (TSS) content in the leaf (**a1**) and root (**a2**) tissues of the BC_4_F_2_ lines with two genetically different backgrounds (“Chinese Spring” (CS) and “Roshan” (R)) under salt stress conditions (250 mM NaCl).

Salt stress significantly affected the total soluble sugar content in the leaves and roots across both line groups (CS and R) (Table 4). The genotypes varied significantly in TSS. Additionally, the interaction of salinity and genotype was significant only in the CS lines. Salinity caused a significant increase in TSS in both leaf and root tissues. Although the increases in TSS in Ac (24.74% in leaves and 46.33% in roots) were lower than those in all wheat lines and cultivars, the leaf and root TSS of Ac were still higher than those of the wheat lines in both environments (Table 5). Under normal conditions, the leaf phenotypes of all selected BC_4_F_2_ lines did not significantly differ from those of their recurrent parent. However, the TSS content in the root tissue of tolerant CS lines was higher than that of their recurrent parent and the sensitive lines. Under stress conditions, the leaf TSS of tolerant CS lines was significantly higher than that of their sensitive counterparts and their recurrent parent. Meanwhile, R lines showed no significant difference between the selected lines and their recurrent parent, except for the leaf of the tolerant line R37 (Supplementary Figure S2). Interestingly, no significant difference was found between the root sugar content in all selected lines and their corresponding parent under stress. Figure 2 shows a strong linear relationship between TSS content in leaf and root tissues and the grain yield of both CS- and R-derived BC_4_F_2_ lines under salinity stress.

#### 3.2.3. Leaf Relative Water Content (RWC)

The results indicated a significant effect of salinity stress, genotype, and the salinity stress × genotype interaction on leaf RWC (Table 4). Salinity stress decreased RWC by about 13% in Ac and up to 33% in wheat (Table 5). The RWC of Ac was consistently higher than that of wheat under both environmental conditions. Under normal conditions, the phenotype of the selected BC_4_F_2_ lines was similar to that of their recurrent parent. Under salinity stress conditions, no significant difference was observed between the RWC of tolerant and sensitive lines derived from CS and R (Supplementary Figure S3).

#### 3.2.4. Malondialdehyde (MDA)

The results showed significant effects of salinity stress, genotype, and the salinity stress × genotype interaction on lipid peroxidation (as measured by MDA content) in leaf tissues (Table 4). While the increase in lipid peroxidation was significant in both wheat and Ac, the change due to salinity was more pronounced in wheat than in Ac. MDA content increased by approximately 40% in Ac and about 140% in wheat due to salinity (Table 5). Notably, the MDA levels in the tolerant lines of both genetic backgrounds were lower than those in their recurrent parents and sensitive lines (Supplementary Figure S4).

#### 3.2.5. DPPH Radical Scavenging Activity

**Figure 3:**
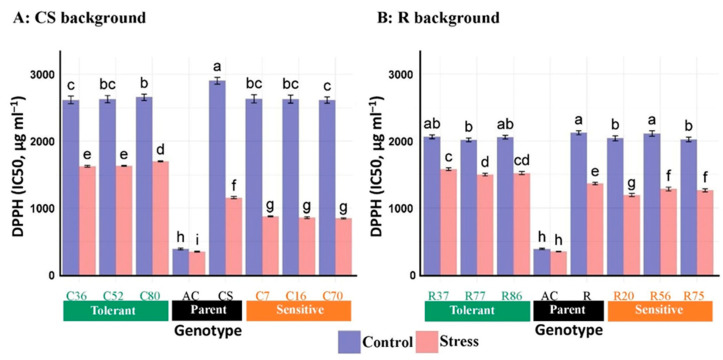
Mean comparison of DPPH radical content, “Chinese spring” (CS), “Roshan” (R) cultivars, *Ae. cylindrica* (AC), and their derived BC_4_F_2_ lines. Bars represent means ± SE, and bars headed by the same letter are not significantly different at *p* ≤ 0.05.

The DPPH scavenging activity was influenced significantly by salinity stress, genotype, and the salinity stress × genotype interaction (Table 4). In normal conditions, the IC50 value for scavenging activity ranged from 389.8 µg mL^−1^ in Ac (the most salt-tolerant genotype) to 2670.7 µg mL^−1^ in CS lines. Likewise, a similar trend was observed in the DPPH scavenging activity under salinity stress conditions (Table 5). The IC50 is the leaf extract concentration needed to scavenge 50% of the initial DPPH radicals. Thus, the DPPH radical scavenging activity of Ac, indicated by a lower IC50 value, was much greater than that of wheat. Although a significant DPPH scavenging activity was observed in all studied genotypes under salinity, it remained consistently higher in wheat. Under normal conditions, the phenotype of all selected genotypes from the CS and R lines with an average of 9.33% and 3.42% showed stronger DPPH scavenging activity than their recurrent wheat parents, respectively. Unexpectedly, all selected tolerant genotypes from CS and R backgrounds exhibited approximately 97.77% and 22.90% higher DPPH radical scavenging activity, respectively, than their sensitive lines under stress conditions. In contrast, the selected sensitive lines CS and R showed on average 25% and 8.5% less DPPH radical scavenging activity, respectively, compared with their recurrent parent lines (Figure 3).

#### 3.2.6. Chlorophyll and Carotenoid Content

Both Chla and Chlb contents were significantly influenced by salinity, genotype, and interaction (Table 4). Compared with normal conditions, salt stress increased by the overall means of Car and Chla, but there was a decrease in Chlb content. The highest changes in Chla (27.92%) and Car (13.55%) due to salt stress were recorded for Ac. In addition, the highest (39.06%) and lowest (28.38%) reductions in Chlb content were reported for the CS lines and Ac, respectively (Table 5). In normal conditions, Chla content in all selected CS and R lines was similar to that of their recurrent parents, except for the sensitive line C7. In salinity stress conditions, the Chla content of all the sensitive lines of the CS background was consistently lower than the tolerant lines (Supplementary Figure S5).

The Chlb content of Ac was higher than that of the studied wheat lines under both normal and salinity conditions. Although the selected CS lines had higher Chlb content than their recurrent parent under normal conditions, this difference was more pronounced in the tolerant lines. In the R lines, most selected lines (except R56) had slightly higher Chlb content than the “Roshan” cultivar under normal conditions. Similarly, in both line groups, the Chlb content of the tolerant lines was significantly higher than that of their recurrent parents and sensitive lines under salinity stress (Supplementary Figure S5). Salinity stress had a minor positive effect on the total chlorophyll content in the tolerant lines of both the CS and R backgrounds. The Car content in Ac under the same conditions was higher than that in the CS lines and was comparable to or slightly higher than in the R lines under both stress and normal conditions. In both genetic backgrounds, the increase in salt-induced Car content was more pronounced in the tolerant lines compared with their sensitive lines and recurrent parents (Supplementary Figure S6).

#### 3.2.7. Na and K Concentrations

**Figure 4:**
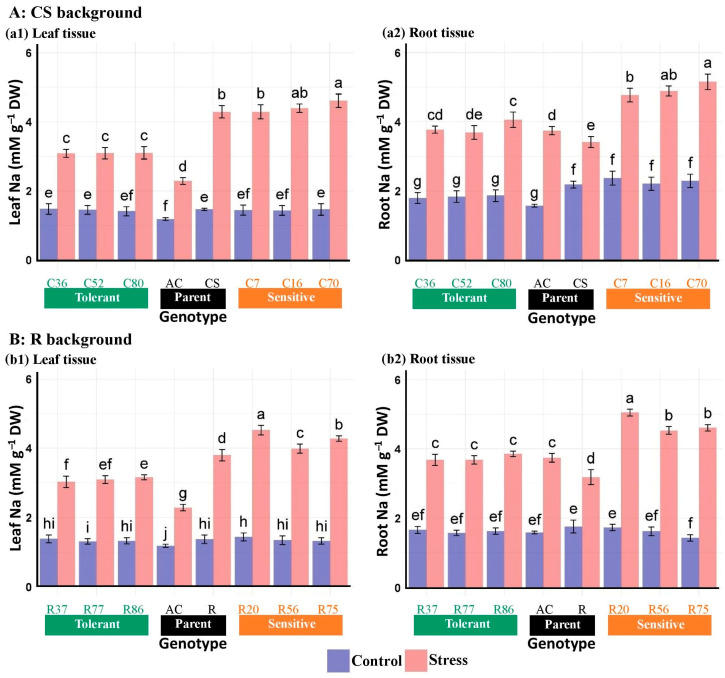
Mean comparison of Na concentration in the leaf and root tissues of “Chinese spring” (CS), “Roshan” (R) cultivars, *Ae. cylindrica* (AC), and their derived BC_4_F_2_ lines. Bars represent means ± SE, and bars headed by the same letter are not significantly different at *p* ≤ 0.05.

The leaf and root Na concentration, K concentration, and K/Na ratio were significantly affected by salinity stress (Table 4). Under salinity stress, Na content increased by 93.8% in Ac leaves, 104.4% in CS roots, 173.1% in R leaves, and 150.8% in R roots compared with normal conditions (Table 5). Under normal conditions, Na accumulated at higher concentrations in the root tissue compared with the leaf tissue across all genotypes. In the CS background, sensitive lines exhibit 28.70% and 43.32% higher sodium concentrations in root and leaf tissue due to salinity compared with tolerant lines. Similarly, in the R background, sensitive lines show a 26.46% and 37.70% increase in sodium concentration in root and leaf tissue, respectively, compared with tolerant lines. Notably, the Na concentration in the root tissue of *Ae. cylindrica* and BC_4_F_2_ lines was higher than the leaves compared with the recurrent wheat cultivars, indicating less sodium transfer to the plant’s aerial organs. In addition, salinity stress resulted in a significantly lower Na accumulation in the root and leaf tissues of tolerant lines compared with sensitive lines (Figure 4).

**Figure 5:**
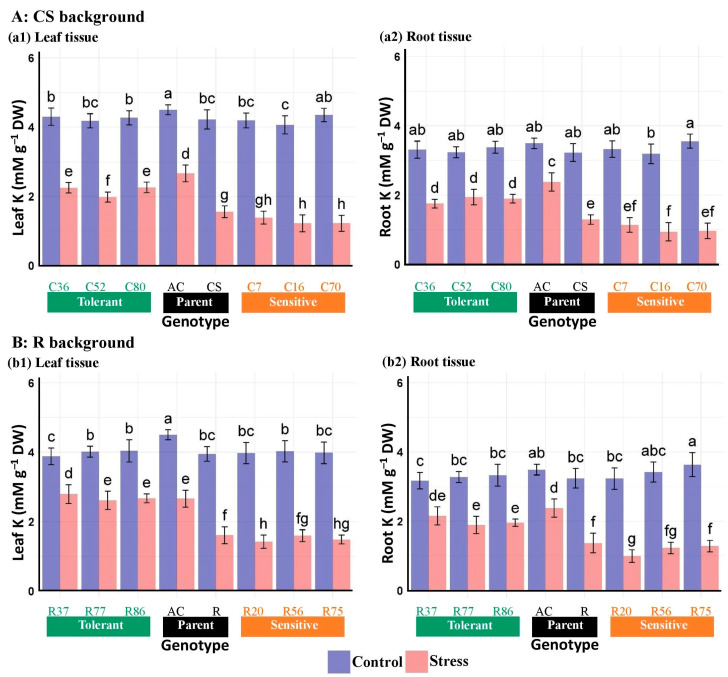
Mean comparison of K concentration in the leaf and root tissues of “Chinese spring” (CS), “Roshan” (R) cultivars, *Ae. cylindrica* (AC), and their derived BC_4_F_2_ lines. Bars represent means ± SE, and bars headed by the same letter are not significantly different at *p* ≤ 0.05.

Salinity stress had a significant inhibitory effect on K concentration, reducing it by 40.88% to 59.94% in leaves and 31.85% to 57.20% in roots across all genotypes (Table 5). However, the K concentration in the leaves was consistently higher than in the roots under both environmental conditions for all genotypes (Figure 5). In addition, the leaf and root K concentrations of *Ae. cylindrica* were higher than those in all studied wheat genotypes under control and stress conditions. Although, salinity resulted in reduced potassium concentrations in the leaves and roots of wheat and *Ae. cylindrica* genotypes, the tolerant lines of both genetic backgrounds maintained higher levels of potassium than their sensitive counterparts (Figure 5).

**Figure 6:**
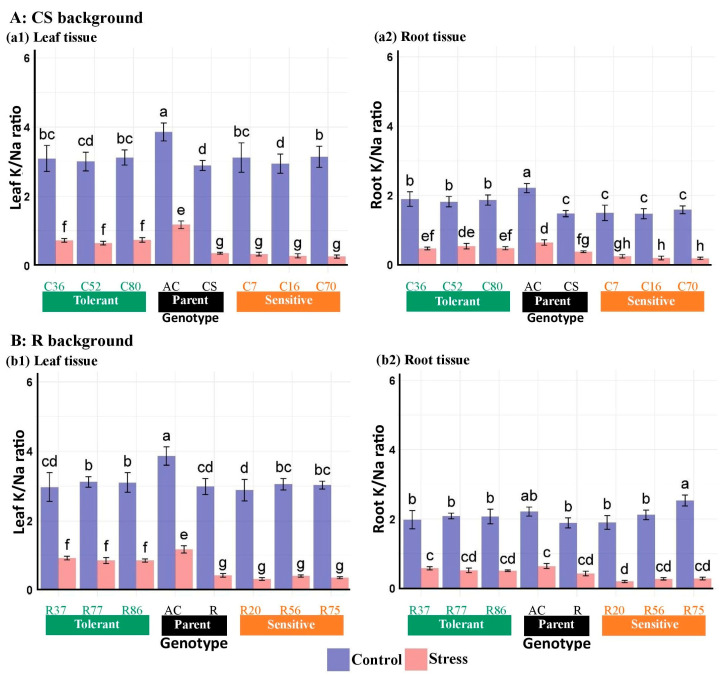
Mean comparison of K/Na content in the leaf and root tissues of “Chinese spring” (CS), “Roshan” (R) cultivars, *Ae. cylindrica* (AC), and their derived BC_4_F_2_ lines. Bars represent means ± SE, and bars headed by the same letter are not significantly different at *p* ≤ 0.05.

Salinity stress significantly reduced the K/Na ratio of leaf and root tissues across all tested genotypes (Figure 6). The reduction was more pronounced in the leaves of the CS lines compared with the roots, while the roots of *A. cylindrica* showed a greater decline than their leaves (Table 5). Comparing the K/Na ratio reduction in CS and R-derived BC_4_F_2_ lines revealed a significant difference between the tolerant and sensitive lines (Figure 6).

**Figure 7:**
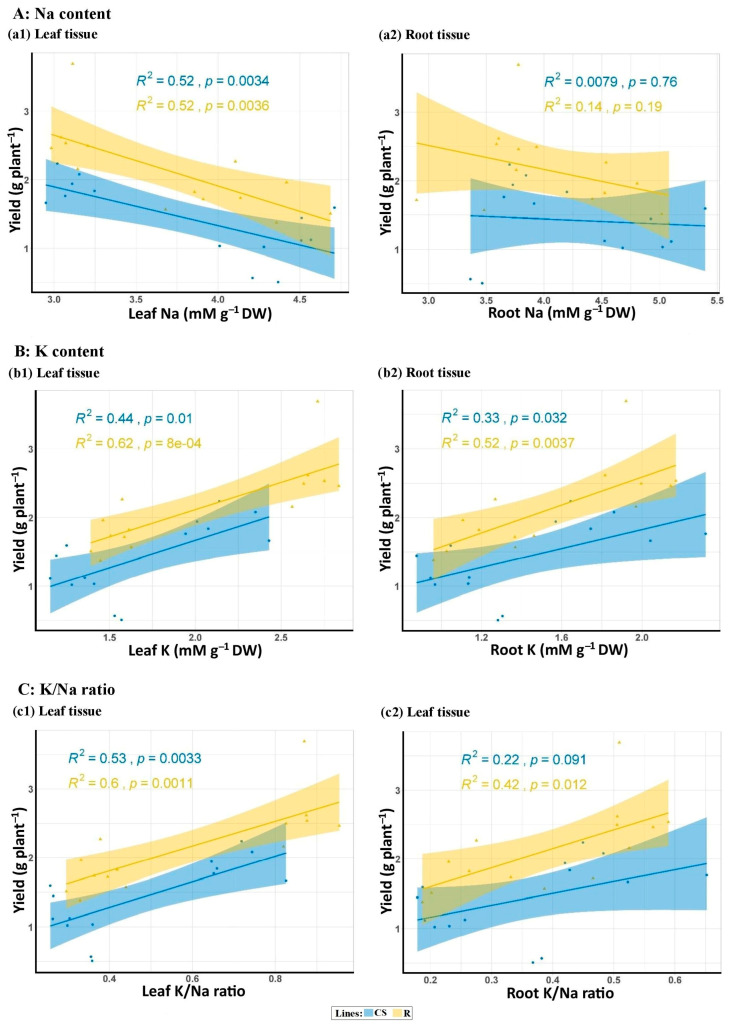
Linear relationship between grain yield (g/plant) and A: Na content, B: K content, and C: K/Na ratio in the leaf (a1, b1, and c1, respectively) and root (a2, b2, c2, respectively) tissues of two groups of BC_4_F_2_ wheat lines with Chinese Spring (CS) and Roshan (R) backgrounds under salt stress conditions (250 mM NaCl).

There was a significant linear relationship between the leaf and root mineral concentrations (Na, K, and K^+^/Na^+^ ratio) and grain yield in both types of BC_4_F_2_ lines, as depicted in Figure 7. For both the R and CS-derived lines, there is a positive and significant relationship between yield and K concentration in both leaf and root tissues. However, the negative relationship between yield and Na concentration was significant only in the leaves of both lines. In addition, the positive relationship of K/Na ratios with yield in leaf tissues is generally significant for both genetic backgrounds. In contrast, this relationship in root tissues varies depending on the genetic background (Figure 7).

### 3.3. DNA Methylation Responses of Introgressed Lines to Salinity

**Figure 8:**
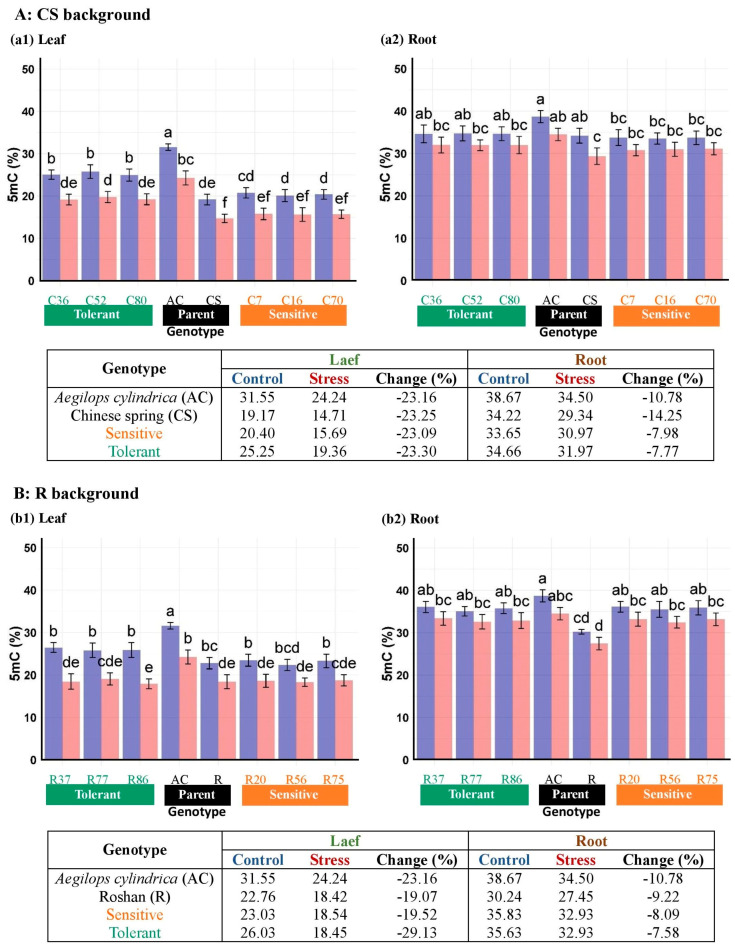
Mean comparison of cytosine methylation levels in the leaf (**a1**,**b1**) and root (**a2**,**b2**) tissues of BC_4_F_2_ lines with “Chinese Spring” CS (**A**); and “Roshan” background (**B**), along with their respective parent (*Ae. cylindrica* (AC), CS, and R). Bars represent means ± SE, and bars headed by the same letter are not significantly different at *p* ≤ 0.05.

The HPLC method determined the 5-mC content of genomic DNA in the leaf and root tissues of BC_4_F_2_ lines and their parents. Salinity stress significantly influenced the 5-mC content of the DNA of the leaf and root tissues of the genotypes (Table 6). In addition, genotypes varied significantly in 5-mC content in the leaves and roots. Moreover, the interactions of salt stress × tissue and genotype × tissue significantly affected 5-mC content. Salinity stress compared with the control treatment leads to a decrease in the 5-mC level in the leaf and root. This stress response was greater in leaf tissue than root tissue in both CS and R line groups. Under control and salinity stress conditions, the DNA methylation level was higher in the root tissue compared with the leaf tissue in all genotypes. Ac exhibited a higher decline in DNA methylation when exposed to salinity than all other wheat genotypes in the leaf and root tissues. Notably, the changes in salinity-induced methylation levels were more in the leaves of tolerant CS and R lines compared with their respective wheat parents and sensitive lines, indicating successful introgression of the *A. cylindrica* epigenome into wheat. In addition, the results suggest that the genetic background influences DNA methylation in different plant organs when exposed to salinity in wheat (Figure 8).

### 3.4. Expression of Salinity Tolerance Genes in the Introgressed Lines

**Figure 9:**
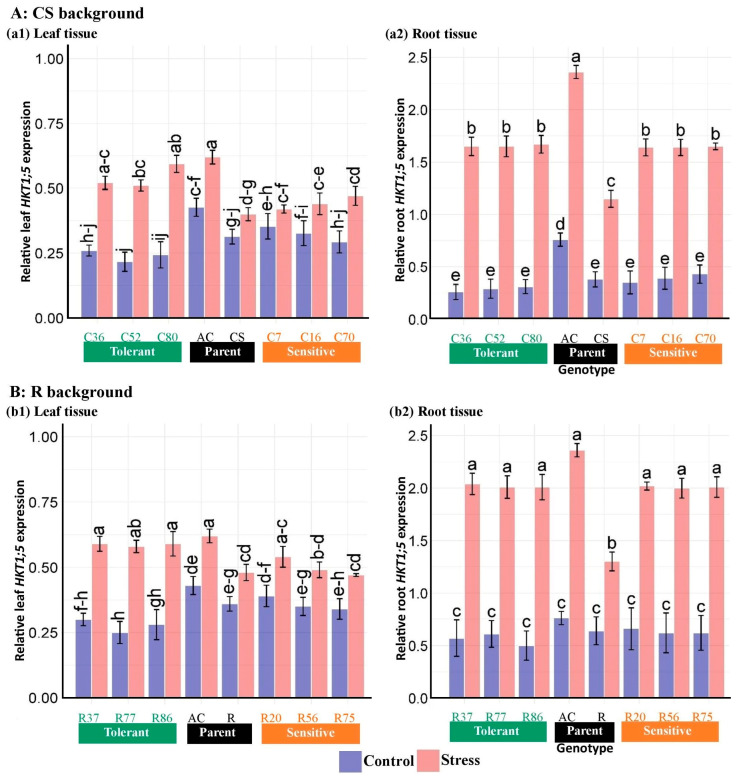
Relative expression analysis of the *HKT1;5* gene in the root and shoot tissues of “Chinese spring” (C), “Roshan” (R) cultivars, *Ae. cylindrica,* and their derived BC_4_F_2_ lines. Bars represent means ± SE, and bars headed by the same letter are not significantly different at *p* ≤ 0.05.

**Figure 10:**
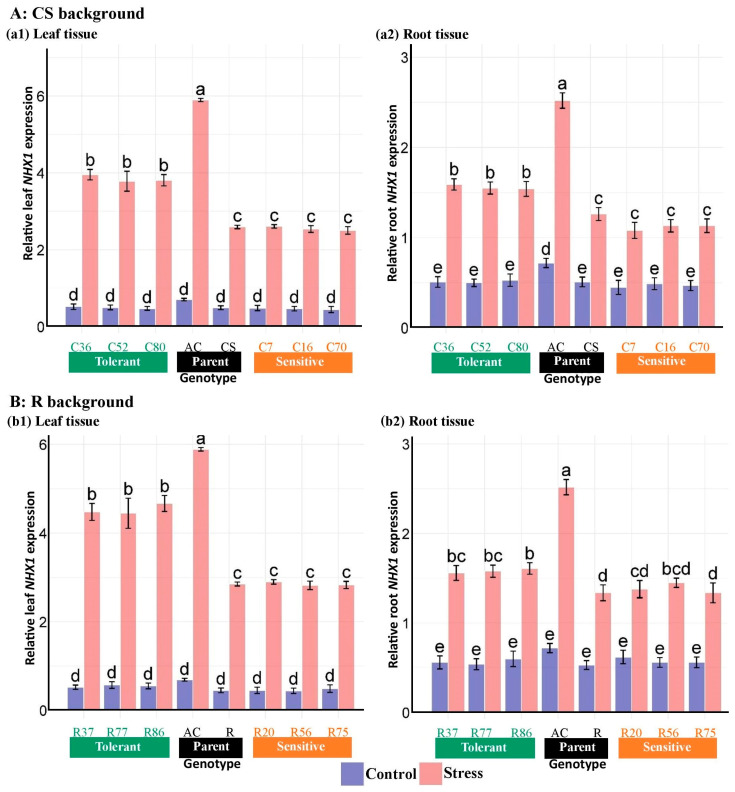
Expression analysis of the *NHX1* gene in the root and shoot tissues of “Chinese spring” (C), “Roshan” (R) cultivars, *Ae. cylindrica,* and their derived BC_4_F_2_ lines. Bars represent means ± SE, and bars headed by the same letter are not significantly different at *p* ≤ 0.05.

**Figure 11:**
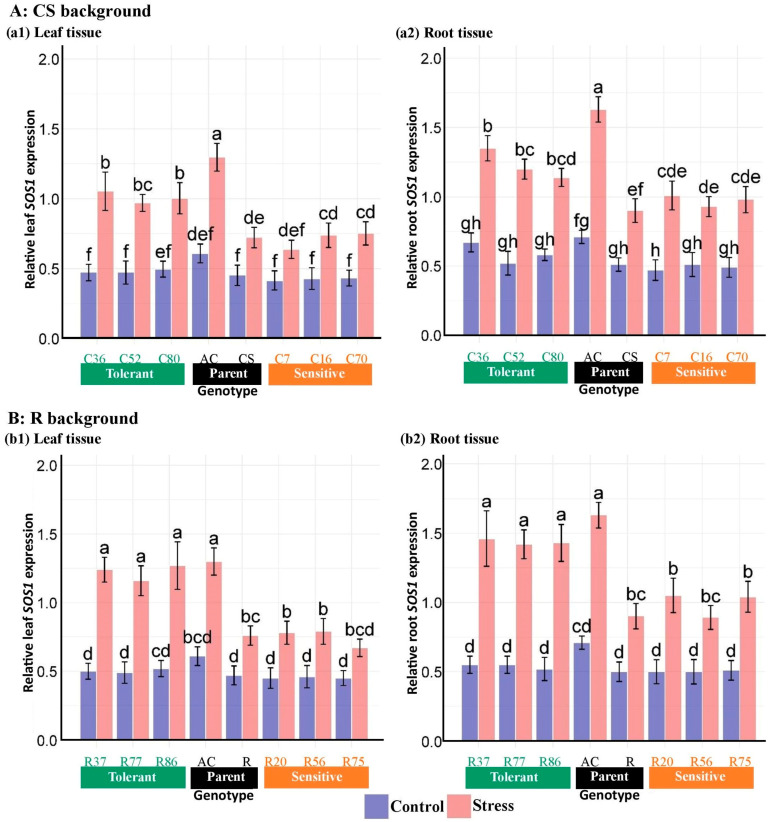
Expression analysis of the *SOS1* gene in the root and shoot tissues of “Chinese spring” (C), “Roshan” (R) cultivars, *Ae. cylindrica,* and their selected BC_4_F_2_ lines. Bars represent means ± SE, and bars headed by the same letter are not significantly different at *p* ≤ 0.05.

ANOVA results of gene expression data showed that salinity, genotype, tissue, and their interactions significantly affected the expression of *HKT1;5*, *NHX1*, and *SOS1* genes in roots and shoots (Table 6). Mean comparisons of the expression patterns of the genes studied indicated that salinity increased the expression level of genes. The expression changes in *HKT1;5* and *SOS1* genes were higher in roots than in leaves in both CS and R-derived, unlike *NHX1*. In the CS lines, the expression level of the *HKT1;5* gene under salinity stress was increased by around 60% in the leaf and around 300% in the root compared with the control (Figure 9). Likewise, changes in *HKT1;5* expression in the R lines were approximately 60 in the leaf and 200% in the root. In contrast, *NHX1* expression alterations were more pronounced in the leaf tissues compared with the roots, with increases of around 580% in the leaf and 180% in the root of the CS lines when exposed to salinity (Figure 10). Similarly, the R lines showed alterations in *NHX1* expression of around 620% in the leaf and 170% in the root. The leaf transcript levels of the *SOS1* gene in the CS and R lines increased by 89% and 95%, respectively, compared with the control, while the root transcripts were upregulated by 104% and 123%, respectively (Figure 11).

At the genotype level, *Ae. cylindrica* had higher *HKT1;5* transcript levels than “Chinese Spring” and “Roshan” in both shoot and root tissues. The *HKT1;5* expression in the root tissues of both BC_4_F_2_ lines did not significantly differ between sensitive and tolerant lines under salinity stress. However, the expression showed a significant increase compared with their wheat parents. Although tolerant genotypes of both line groups generally expressed higher *HKT1;5* levels than their sensitive counterparts in the shoots, this difference was not significant in most cases (Figure 9).

Under stress conditions, the *NHX1* expression level of the salt-tolerant Ac in both tissues was significantly higher than in the lines and cultivars. Although comparable mRNA expression levels for *NHX1* were not observed between tolerant R and CS lines in the root tissue under stress, this comparison was evident in the leaf tissue under salinity, with noticeably higher *NHX1* expression levels in the tolerant R lines than in the tolerant CS lines. On the other hand, the leaves and roots of sensitive R lines showed higher *NHX1* expression levels than the sensitive CS lines under stress conditions. Interestingly, despite no significant difference in *NHX1* expression between sensitive and tolerant lines under control conditions, *NHX1* transcript levels in both tissues of tolerant lines from both genetic backgrounds were significantly higher than their wheat parents and sensitive lines under salinity (Figure 10).

The *SOS1* transcripts of the salt-tolerant Ac genotype in both tissues under salinity significantly exceeded all CS lines. However, the mRNA expression level for the *SOS1* gene in both tissues of all tolerant CS lines was recorded as higher than wheat parent in stress conditions. In addition, the *SOS1* expression levels of tolerant R-lines were non-significantly with the salt-tolerant Ac genotype in both tissues under stress. However, the induction of *SOS1* gene expression did not significantly differ between sensitive genotypes and recurrent wheat parents in studied tissues under salt stress. The increase in the *SOS1* expression level was more pronounced in the root tissue of sensitive lines compared with their leaf tissue. Therefore, at the tissue level, increased expression of *SOS1* in leaf tissue is more crucial than in roots for salt stress tolerance (Figure 11).

## 4. Discussion

Plants manage salinity through mechanisms like osmoregulation, oxidative damage protection, and maintaining low cytosolic sodium levels via sodium exclusion, intracellular compartmentalization, and K^+^/Na^+^ homeostasis [53,54]. Wild wheat relatives from the Fertile Crescent, adapted to abiotic stress through natural selection, are valuable for introgressing adaptive traits and enhancing vitality [1,55,56].

Grain yield links plant biochemical responses to stressors and adaptation through genetic [1] and epigenetic diversity [28]. Genetic diversity in wheat arises from DNA variation and epigenetic modulation, leading to differential morpho-physiological responses. Our study shows significant genotype and genotype-by-stress interaction effects on grain yield, with considerable variation in salinity responses among wheat-introgressed lines. Notably, *Ae. cylindrica* (AC) and introgressed lines were less affected by salinity than recurrent wheat parents, confirming successful Ac genome introgression and supporting further physiological and molecular studies.

Salinity significantly increased proline content in leaves, especially in Ac, with lower responses in R background lines, while roots showed a weaker response. These results help explain conflicting reports on proline’s role in salt tolerance, influenced by species, genotype, tissue type, growth stage, and salinity intensity. Proline’s positive effects on salt adaptation, mainly through osmotic potential balance in intracellular compartments, are evident under low to moderate salinity stress or early exposure stages [57]. However, some studies suggest its role is more critical in drought conditions [7]. Arabbeigi et al. [7] found that the *P5CS* gene enhances proline biosynthesis in Ac species, yet proline did not significantly contribute to salt tolerance. Our findings align with Kumar et al. [58], who reported proline’s positive effect on salinity tolerance in wheat, emphasizing the genetic influence on proline’s role in stress tolerance. This is partially consistent with Ebrahim et al. [22], who observed higher proline accumulation in salt-sensitive barley genotypes than salt-tolerant ones. Additionally, the more drastic proline changes in Ac compared with wheat align with Munns and Tester’s review [54]. Compared with roots, the higher proline accumulation in leaves under salinity stress is justified by the leaves’ crucial role in gas exchange and osmotic balance maintenance, protecting the photosynthetic apparatus in these metabolically active tissues.

Increased soluble sugars in leaves align with previous observations [15,58,59,60], while root accumulation shows mixed results [15,58,60]. Lower accumulation of sugars in roots compared with leaves likely stems from leaves being primary photosynthesis sites and factors similar to those affecting proline. Inconsistencies in osmo-adaptive responses between plant organs can be attributed to species, genotype, stress intensity, duration, and growth stage differences [22]. Leaves accumulate osmoprotectants like proline and TSS to combat osmotic stress, maintain cellular water potential, and protect cellular components.

MDA content, an indicator of lipid peroxidation, increased most in sensitive wheat cultivars (“Chinese Spring” and “Roshan”) and their derived lines, with the lowest increase in the donor parent (Ac) and tolerant lines. Lower lipid peroxidation levels in salt-tolerant AC genotypes [16], their amphidiploids (AC × wheat) [9], and salt-tolerant wheat cultivars [58] align with our findings. Similarly, Zeeshan et al. [61] reported significant MDA content changes under salt stress, particularly in sensitive cultivars. In wheat, MDA content negatively correlates with membrane stability [58,62]. MDA, generated by ROS-induced oxidative stress, leads to lipid peroxidation in cell membranes, and measuring MDA levels is a marker for assessing cell membrane stability and disruptions in osmotic balance under stress [62,63].

DPPH, a stable free radical, assesses antioxidant activity by evaluating free radical scavenging and lipid oxidation inhibition. A lower IC50 value indicates higher antioxidant capacity. Our study found the highest DPPH inhibition (lowest IC50 value) in the donor wild parent of the BC_4_F_2_ lines (Ac), a hyper-salt-tolerant genotype [16], followed by R background lines and then CS lines. These results highlight the influence of wheat genetic makeup on the antioxidant activity of the BC_4_F_2_ lines, consistent with studies emphasizing the genetic diversity and strong DPPH scavenging ability of salt-tolerant genotypes [9,58,64,65,66,67,68]. These findings suggest DPPH’s involvement in wheat’s antioxidant defense against salt stress.

Salt stress increased Car and Chla means and decreased Chlb in wheat lines and Ac, with contrasting effects on total chlorophyll content. The decrease in total chlorophyll in the CS background and the increase in the R background highlight salinity tolerance differences. This finding aligns with Kiani et al. [9], who reported a non-significant decrease in total Chl content in tolerant genotypes (amphidiploids) compared with a significant decrease in wheat cultivars. Differences in chlorophyll content under salinity have been reported in wheat [58,69].

Plants cope with sodium toxicity through selective ion transporters to exclude Na^+^ from the cytosol. However, when these defenses are insufficient, Na^+^ toxicity can lead to significant cellular damage and reduced plant growth. Our study found significantly lower leaf Na concentrations in Ac, a halophyte [24], compared with other genotypes, under salinity stress. Tolerant BC_4_F_2_ lines derived from CS and R ranked second in reduced Na accumulation in leaf tissues. At the same time, no significant root Na concentration differences were found between the introgressed lines and their donor parent (Ac), contrasting with the recurrent parents “Chinese Spring” and “Roshan” cultivars. The higher Na accumulation in roots compared with leaves under salinity stress can be explained by the ability to sequester Na^+^ in root vacuoles; improved Na^+^ retrieval from leaves and transport back to the roots; and decreased Na^+^ unloading from xylem in roots [70]. Evidence supporting this hypothesis includes higher *HKT1;5* gene transcript abundance in roots than in leaves. Similar findings in barley [71] and wheat [11] show higher *HKT1;5* expressions in roots compared with shoots, with expression levels inversely correlated with Na^+^ concentration. *TaHKT1;5-D* expression in roots is specifically found in the xylem parenchyma and pericycle cells next to the xylem cells within the stele [72]. The *HKT1;5* gene in *T. aestivum* functions as a selective Na^+^ transporter, unloading Na^+^ from xylem vessels to parenchymal cells [73,74]. Recent data indicate *HKT1;5* alleles are genetically associated with Na^+^ accumulation in shoots and salinity tolerance [75].

This study examined *HKT1;5*, *NHX1*, and *SOS1* gene expression in selected BC_4_F_2_ lines derived from amphidiploid plants (R × Ac and CS × Ac). The lines reacted differently than the wheat cultivars (recurrent parents), comparable to or inferior to their donor parent (AC). These findings are consistent with agro-physiological and biochemical analysis. Differences in salt stress responses between lines from contrasting wheat cultivars underscore the genetic background’s influence on stress responses. Coordination at the transcriptional level was observed among *HKT1;5*, *NHX1*, and *SOS1*, with expression levels following the order: Ac > lines > wheat cultivars. This suggests that salt stress may act as a synchronous environmental stimulus, reflected in the divergence and specificity of induced molecular signatures. The relative activation, repression, contribution, and magnitude of involved transcripts may determine the specificity of salt stress adaptation. The synergistic function of these genes aligns with other studies [26,57]. Co-regulation of *HKT1;5*, *NHX1*, and *SOS1* may be crucial for fine-tuning wheat’s salinity tolerance mechanism. In contrast to *HKT1;5*, higher *NHX1* expression in leaves than in roots is consistent with other studies [11,76]. The inverse tissue-specific functionality of *NHX1* and *HKT1;5* suggests neofunctionalisation and partial subfunctionalisation of homoeologous ion transporter genes during wheat allopolyploidisation.

Salt stress reduced K concentration most in salt-sensitive lines and their wheat parents, affecting leaf and root tissues. Salt-tolerant wheat lines and Ac maintained a higher leaf K/Na ratio under stress, while salt-sensitive genotypes were less effective. Dual-affinity Na^+^ transporters encoded by *TaHKT1;5-D* and *TmHKT1;5-A* genes are inhibited by increasing external K^+^ concentrations [77]. Higher *HKT1;5* transcription in *AC* and its derived salt-tolerant lines may involve epigenetic modulation. Differences in promoter strength, transcription factor availability, gene copy number, regulatory elements, and RNA stability cannot be ruled out.

Salt tolerance involves genetic and epigenetic factors [78]. Epigenetic alterations modify gene transcription, aiding adaptation to abiotic stresses and providing “plant stress memory” for improved environmental responses [28]. Epigenetic mechanisms like DNA methylation, histone modifications, chromatin remodeling, epi-transcriptomics, and small RNA-mediated gene silencing regulate gene expression, which is crucial for adaptation and phenotypic plasticity [28,79]. Cytosine methylation at the 5ʹ position (m5C) is the most abundant DNA modification, affecting up to 25% of cytosines in the plant genome [80]. Global DNA methylation analysis showed a notable difference in cytosine methylation content between AC and wheat under salinity stress, highlighting the genetic background’s effect on methylation and demethylation events in wheat introgression lines.

Compared with their recurrent wheat parents, the salinity-induced epigenetic variations detected in *AC* and its derived wheat lines may play a role in adaptation to salinity stress. However, further studies are needed to clarify the roles of small RNAs, histone post-translational modifications, and chromatin remodeling in this context. Differences in the regulatory epigenetic landscape may contribute to adaptive molecular responses, which can be manipulated to accelerate plant breeding programs. Selective cross-breeding and epigenome modulation can enhance wheat’s adaptive potential to salinity stress in the face of climate change. Genetic resources from wild relatives of cereals and other crops, particularly in the Fertile Crescent, have successfully introduced valuable genes to cultivated cereals. Our study demonstrates that wild *AC* can improve salinity tolerance in cultivated wheat lines, which is consistent with other studies emphasizing the potential of wild species for enhancing crop resilience.

## 5. Conclusions

In addition to the necessity of genetic diversity for improving crop plants, the breeding implications of epigenomic diversity are crucial. Consistent cytosine methylations at so-called epialleles in a particular genotype can serve as selection markers for abiotic stress tolerance in breeding programs. This study linked agro-physiological traits with epigenetic marks to confidently identify the most contrasting salt-responsive introgressed wheat lines. A greater reduction in global methylation content and a significant increase in *HKT1;5*, *NHX1*, and *SOS1* gene expression in the leaves of tolerant lines compared with sensitive lines under salinity stress highlight the important role of methylation buffering in adaptation through gene expression regulation. Further studies are needed to better understand the functional underpinnings of how the DNA methylation affects stress tolerance and the inheritance of epialleles in plants under stressful environments.

## Figures and Tables

**Table 1:** Results of analysis of variance for the agronomic traits studied in 73 (72 CS-derived BC_4_F_2_ lines and CS cv) and 85 (54 R-derived BC_4_F_2_ lines and “Roshan” cv) genotypes of wheat grown under control and salt-stress (250 mM NaCl) field conditions.

Source of Variation	df	Mean Square					
		Grain Yield	Grain Weight	Grain per Spike	Spikes Per Plant	Spike Length	Plant Height
“Chinese Spring” background							
Year (Y)	1	2.77 **	1.53 **	408.76 **	228.32 **	1.99 ^ns^	74,324.90 **
Stress (S)	1	187.41 **	78.41 **	13,386.65 **	31.72 **	73.58 **	1846.71 **
Genotype (G)	72	2.31 **	1.23 **	157.43 **	22.43 **	2.75 **	160.18 **
Y × S	1	0.04 ^ns^	0.43 ^ns^	456.56 **	4.69 ^ns^	0.09 ^ns^	62.15 ^ns^
Y × G	72	0.03 ^ns^	0.04 ^ns^	1.52 ^ns^	7.7 **	0.07 ^ns^	117.27 **
S × G	72	1.42 **	0.63 **	78.65 **	14.38 **	0.77 ^ns^	12.48 ^ns^
Y × S × G	72	0.02 ^ns^	0.01 ^ns^	2.99 ^ns^	9.20 **	0.02 ^ns^	5.48 ^ns^
Residual	576	0.08	0.14	15.01	1.59	1.57	37.75
CV (%)		20.87	17.66	17.20	37.09	16.1	8.50
“Roshan” background							
Year (Y)	1	0.06 ^ns^	5.94 **	18.46 ^ns^	0.83 ^ns^	1.53 ^ns^	2211.56 **
Stress (S)	1	448.59 **	26.12 **	18,897.31 **	48.48 **	237.16 **	99,573.21 **
Genotype (G)	84	2.95 **	1.84 **	125.26 **	3.11 **	1.73 ^ns^	137.00 **
Y × S	1	5.77 **	3.00 *	324.32 **	7.07 **	17.14 **	1.96 ^ns^
Y × G	84	0.16 ^ns^	0.25 ^ns^	15.05 ^ns^	0.35 ^ns^	0.43 ^ns^	13.99 ^ns^
S × G	84	1.76 **	1.20 **	114.50 **	2.18 **	1.86 ^ns^	108.68 **
Y × S × G	84	0.05 ^ns^	0.15 ^ns^	9.48 ^ns^	0.10 ^ns^	0.86 ^ns^	28.68 ^ns^
Residual	672	0.28	0.60	44.94	0.51	1.70	46.12
CV (%)		23.62	22.79	22.21	31.07	12.72	8.99

The symbols along the rows and columns indicate: ^ns^ Non-significant; * *p* ≤ 0.05, ** *p* ≤ 0.01.

**Table 2:** Coefficient correlations between yield, grain weight (GW), number of grains per spike (GpS), number of spikes per plant (SpP), spike length (SL), and plant height (PH) under normal (above diagonal) and salinity stress (below diagonal) field conditions. The trait names are shown in the diagonal.

“Chinese Spring” Background					
Yield	0.29 ***	0.39 ***	0.37 ***	0.18 ***	0.32 ***
0.37 ***	GW	0.20 ***	−0.13 ***	0.40 ***	0.38 ***
0.35 ***	0.50 ***	GpS	−0.15 ***	0.31 ***	0.21 ***
0.44 ***	−0.40 ***	−0.49 ***	SpP	−0.13 **	0.04
0.20 ***	0.57 ***	0.48 ***	−0.34 ***	SL	0.57 ***
0.33 ***	0.60 ***	0.50 ***	−0.26 ***	0.72 ***	PH
“Roshan” Background					
Yield	0.14 **	0.08	0.72 ***	0.27 ***	0.30 ***
−0.23 ***	GW	−0.38 ***	−0.15 ***	0.22 ***	0.22 ***
0.52 ***	−0.10 *	GpS	−0.34 ***	0.44 ***	0.22 ***
0.61 ***	−0.65 ***	−0.09 *	SpP	−0.18 ***	−0.02
0.48 ***	−0.07	0.34 ***	0.20 ***	SL	0.64 ***
0.64 ***	−0.15 ***	0.40 ***	0.34 ***	0.64 ***	PH

The symbols along the rows and columns indicate: * *p* ≤ 0.05, ** *p* ≤ 0.01; *** *p* ≤ 0.001.

**Table 3:** Means * of grain yield (g plant^−1^) of selected introgressed wheat lines derived from wheat × *Ae. cylindrica* (“Chinese Spring” as the recurrent parent and “Roshan” as the recurrent parent) under normal (Yn) and salinity stress (Ys) conditions as well as selection indices over two years.

Background	Group	Genotypes	Years	Yn	Ys	YSI	YI	HM	STI	GMP	MRP	MP	TOL	YLI	SSI
Chinese Spring (CS)	Sensitive	C7	1	3.19	1.04	0.32	1.14	1.56	1.00	1.82	2.90	2.11	2.16	0.68	1.35
			2	3.46	1.12	0.32	1.12	1.70	1.03	1.97	2.90	2.29	2.34	0.68	1.40
		C16	1	3.65	1.02	0.28	1.13	1.60	1.13	1.93	3.14	2.34	2.63	0.72	1.44
			2	4.06	1.12	0.28	1.11	1.75	1.20	2.13	3.20	2.59	2.94	0.72	1.50
		C70	1	3.91	1.44	0.37	1.60	2.11	1.71	2.38	3.75	2.68	2.47	0.63	1.26
			2	4.13	1.59	0.39	1.59	2.30	1.74	2.57	3.72	2.86	2.54	0.61	1.27
	Recurrent parent	Chinese spring	1	2.15	0.51	0.24	0.56	0.82	0.33	1.05	1.75	1.33	1.64	0.76	1.53
			2	2.43	0.57	0.23	0.57	0.92	0.37	1.17	1.82	1.50	1.86	0.77	1.59
	Tolerant	C36	1	2.39	2.24	0.94	2.47	2.31	1.62	2.31	3.79	2.31	0.15	0.06	0.12
			2	2.58	2.08	0.81	2.07	2.30	1.42	2.32	3.40	2.33	0.50	0.19	0.40
		C52	1	2.12	1.94	0.92	2.15	2.03	1.25	2.03	3.31	2.03	0.17	0.08	0.16
			2	2.31	1.77	0.76	1.76	2.00	1.08	2.02	2.95	2.04	0.54	0.24	0.49
		C80	1	1.94	1.66	0.86	1.84	1.79	0.98	1.79	2.90	1.80	0.27	0.14	0.28
			2	2.23	1.84	0.82	1.83	2.02	1.09	2.03	2.98	2.04	0.39	0.18	0.37
Roshan (R)	Sensitive	R20	1	5.50	1.38	0.25	0.91	2.20	0.85	2.75	2.76	3.44	4.13	0.75	1.51
			2	5.58	1.51	0.27	0.92	2.38	1.06	2.90	2.90	3.55	4.07	0.73	1.75
		R56	1	4.59	1.82	0.40	1.21	2.61	0.94	2.89	2.75	3.21	2.77	0.60	1.22
			2	4.43	2.27	0.51	1.38	3.00	1.27	3.17	2.95	3.35	2.16	0.49	1.17
		R75	1	4.87	1.73	0.36	1.15	2.56	0.95	2.91	2.78	3.30	3.13	0.64	1.30
			2	4.94	1.96	0.40	1.20	2.81	1.22	3.11	2.95	3.45	2.97	0.60	1.44
	Recurrent parent	Roshan	1	3.49	1.57	0.45	1.04	2.16	0.62	2.34	2.21	2.53	1.93	0.55	1.11
			2	3.25	1.72	0.53	1.05	2.25	0.70	2.36	2.20	2.48	1.53	0.47	1.13
	Tolerant	R37	1	2.94	2.46	0.84	1.64	2.68	0.81	2.69	2.62	2.70	0.48	0.16	0.33
			2	2.59	2.54	0.98	1.55	2.56	0.83	2.56	2.47	2.56	0.05	0.02	0.05
		R77	1	3.16	2.62	0.83	1.74	2.86	0.93	2.87	2.80	2.89	0.54	0.17	0.35
			2	2.93	2.16	0.74	1.32	2.49	0.80	2.52	2.36	2.55	0.78	0.26	0.63
		R86	1	4.36	3.69	0.85	2.45	4.00	1.81	4.01	3.92	4.03	0.67	0.15	0.31
			2	3.20	2.50	0.78	1.52	2.80	1.01	2.83	2.66	2.85	0.71	0.22	0.53

* Different colors represent two groups of lines: CS (red) and R (blue) genetic backgrounds. The intensity of each color indicates the value of a particular trait, with stronger colors signifying higher values.

**Table 4:** Results of analysis of variance for the physiological traits studied under control and salt-stress (250 mM NaCl) treatments.

Source of Variation	df	Mean Square																
		Proline (Leaf)	Proline (Root)	TSS (Leaf)	TSS (Root)	RWC (Leaf)	MDA (Leaf)	DPPH (Leaf)	Chla	Chlb	Total chl	Car	Na (Leaf)	Na (Root)	K (Leaf)	K (Root)	K/Na (Leaf)	K/Na (Root)
“Chinese Spring” background																		
Year (Y)	1	1.36 ^ns^	0.53 ^ns^	0.02 ^ns^	0.10 ^ns^	0.15 ^ns^	0.14 ^ns^	5310.82 ^ns^	0.03 ^ns^	0.003 ^ns^	0.01 ^ns^	1.33 ^ns^	0.30 ^ns^	0.53 ^ns^	0.44 ^ns^	0.02 ^ns^	0.99 ^ns^	0.19 ^ns^
Stress (S)	1	910.93 *	100.99 *	130.26 *	84.97 *	14,095.71 *	541.9 *	37,755,458.59 *	1.01 *	1.38 **	0.03 ^ns^	5.33 *	119.91 *	113.02 *	143.47 *	77.69 *	160.16 *	42.91 *
Genotype (G)	7	76.1 **	5.15 **	23.97 **	19.84 **	383.18 **	11.21 **	4,195,117.78 **	0.027 **	0.036 **	0.12 **	1.25 **	2.52 **	2.27 **	1.22 **	0.96 **	1.03 **	0.54 **
Y × S	1	0.74 ^ns^	0.19 ^ns^	0.19 ^ns^	0.17 ^ns^	82.80 ^ns^	0.61 ^ns^	33,227.79 ^ns^	0.005 ^ns^	0.00 ^ns^	0.00 ^ns^	0.02 ^ns^	0.04 ^ns^	0.07 ^ns^	0.14 ^ns^	0.45 ^ns^	0.67 ^ns^	0.29 ^ns^
Y × G	7	0.04 ^ns^	0.01 ^ns^	0.18 ^ns^	0.25 ^ns^	26.02 ^ns^	0.09 ^ns^	66.03 ^ns^	0.00 ^ns^	0.00 ^ns^	0.00 ^ns^	0.00 ^ns^	0.04 ^ns^	0.06 ^ns^	0.02 ^ns^	0.11 ^ns^	0.01 ^ns^	0.02 ^ns^
S × G	7	50.18 **	1.22 **	3.58 **	1.76 **	225.18 **	13.89 **	1,163,351.31 **	0.01 **	0.001 *	0.02 **	0.58 **	1.89 **	0.79 **	0.67 **	0.80 **	0.13 **	0.06 *
Y × S × G	7	0.05 ^ns^	0.01 ^ns^	0.18 ^ns^	0.23 ^ns^	25.95 ^ns^	0.17 ^ns^	814.53 ^ns^	0.00 ^ns^	0.00 ^ns^	0.00 ^ns^	0.01 ^ns^	0.036 ^ns^	0.04 ^ns^	0.02 ^ns^	0.06 ^ns^	0.01 ^ns^	0.01 ^ns^
Residual	56	0.73	0.65	0.31	0.36	13.09	0.78	9786.05	0.03	0.01	0.04	0.42	0.16	0.21	0.34	0.34	0.34	0.10
CV (%)		13	18.48	11.06	15.33	5.21	14.81	5.62	14.62	20.49	11.52	6.4	15.85	14.85	19.33	24.03	31.60	29.86
“Roshan” background																		
Year (Y)	1	0.44 ^ns^	0.09 ^ns^	0.06 ^ns^	0.49 ^ns^	206.08 ^ns^	1.49 ^ns^	19,993.05 ^ns^	0.07 ^ns^	0.00 ^ns^	0.04 ^ns^	1.18 ^ns^	0.40 ^ns^	0.09 ^ns^	0.32 ^ns^	0.00 ^ns^	0.83 ^ns^	0.19 ^ns^
Stress (S)	1	491.53 *	33.81 *	140.72 *	75.4 **	16,432.48 *	699.58 *	8,533,011.28 *	1.81 *	1.27 *	0.04 ^ns^	7.53 *	114.75 *	140.53 *	90.44 *	68.84 **	145.99 *	67.48 *
Genotype (G)	7	85.77 **	5.53 **	17.94 **	14.67 **	339.34 **	12.71 **	2,809,371 **	0.01 **	0.03 **	0.03 **	0.63 **	1.95 **	1.09 **	1.50 **	0.79 *	1.03 **	0.19 ^ns^
Y × S	1	0.31 ^ns^	0.06 ^ns^	0.17 ^ns^	0.007 ^ns^	24.22 ^ns^	0.80 ^ns^	24,740.43 ^ns^	0.00 ^ns^	0.00 ^ns^	0.01 ^ns^	0.03 ^ns^	0.05 ^ns^	0.21 ^ns^	0.06 ^ns^	0.01 ^ns^	0.47 ^ns^	0.22 ^ns^
Y × G	7	0.02 ^ns^	0.07 ^ns^	1.04 ^ns^	0.88 ^ns^	25.82 ^ns^	0.05 ^ns^	443.90 ^ns^	0.00 ^ns^	0.00 ^ns^	0.00 ^ns^	0.01 ^ns^	0.01 ^ns^	0.12 ^ns^	0.00 ^ns^	0.12 ^ns^	0.00 ^ns^	0.11 ^ns^
S × G	7	63.36 **	1.23 **	1.12 ^ns^	0.71 ^ns^	261.04 **	12.78 **	215,121.07 **	0.00 ^ns^	0.01 **	0.02 **	0.54 **	1.46 **	1.27 **	1.06 **	0.86 **	0.17 **	0.23 *
Y × S × G	7	0.01 ^ns^	0.02 ^ns^	1.02 **	0.75 ^ns^	28.40 ^ns^	0.05 ^ns^	2515.14 ^ns^	0.00 ^ns^	0.00 ^ns^	0.00 ^ns^	0.02 ^ns^	0.01 ^ns^	0.03 ^ns^	0.00 ^ns^	0.04 ^ns^	0.00 ^ns^	0.07 ^ns^
Residual	56	0.34	0.43	0.26	0.36	20.33	0.20	4540.07	0.02	0.006	0.02	0.61	0.09	0.09	0.30	0.37	0.25	0.10
CV (%)		9.58	15.51	9.60	14.25	6.41	6.43	4.33	10.30	15.07	8.61	7.51	12.73	10.59	17.78	24.28	26.50	25.35

Trait abbreviations: (total soluble sugars), RWC (relative water content), MDA (malondialdehyde), DPPH (2,2-diphenyl-1-picrylhydrazyl), Chla (chlorophyll a), Chlb (chlorophyll b), total chl (total chlorophyll), Car (carotenoid), Na and K concentrations and K/Na ratios. The symbols along the rows and columns indicate: ^ns^ Non-significant; * *p* ≤ 0.05, ** *p* ≤ 0.01.

**Table 5:** The mean performance of the physiological trait of *Ae. cylindrica* and two genetic background lines of wheat (CS and R) under control and salinity stress (250 mM NaCl) conditions.

Trait	*Ae. cylindrica*			CS Derived Lines			R Derived Lines		
	Control (0 mM NaCl)	Stress (250 mM NaCl)	Change (%)	Control (0 mM NaCl)	Stress (250 mM NaCl)	Change (%)	Control (0 mM NaCl)	Stress (250 mM NaCl)	Change (%)
Leaf Proline	4.81 ^b^	20.63 ^a^	328.85	3.33 ^b^	8.12 ^a^	143.38	3.71 ^b^	6.63 ^a^	78.39
Root Proline	4.51 ^b^	7.24 ^a^	60.53	3.18 ^b^	5.13 ^a^	61.51	3.54 ^b^	4.51 ^a^	27.29
Leaf TSS	7.35 ^b^	9.17 ^a^	24.74	3.40 ^b^	5.80 ^a^	70.74	3.68 ^b^	6.19 ^a^	68.07
Root TSS	5.62 ^a^	8.22 ^a^	46.33	2.58 ^b^	4.36 ^a^	68.98	3.01 ^b^	4.66 ^a^	54.94
RWC	86.96 ^a^	75.41 ^a^	−13.29	80.70 ^a^	54.65 ^b^	−32.28	82.86 ^a^	54.60 ^b^	−34.10
MDA	4.01 ^b^	5.62 ^a^	40.18	3.54 ^b^	8.74 ^a^	146.93	4.27 ^b^	10.21 ^a^	138.97
DPPH	389.80 ^a^	350.10 ^b^	−10.18	2670.70 ^a^	1242.94 ^b^	−53.46	2062.30 ^a^	1386.51 ^b^	−32.77
Chla	1.13 ^b^	1.45 ^a^	27.92	1.10 ^b^	1.29 ^a^	17.33	1.21 ^b^	1.48 ^a^	22.15
Chlb	0.73 ^a^	0.53 ^b^	−28.38	0.63 ^a^	0.38 ^b^	−39.06	0.63 ^a^	0.40 ^b^	−36.84
Total chl	1.87 ^b^	1.97 ^a^	5.76	1.72 ^a^	1.67 ^a^	−3.16	1.85 ^a^	1.88 ^a^	1.89
Car	10.23 ^a^	11.61 ^a^	13.55	9.92 ^b^	10.26 ^a^	3.44	10.14 ^b^	10.58 ^a^	4.36
Leaf Na	1.18 ^b^	2.29 ^a^	93.82	1.44 ^b^	3.84 ^a^	166.44	1.35 ^b^	3.69 ^a^	173.06
Root Na	1.58 ^b^	3.74 ^a^	136.28	2.08 ^b^	4.25 ^a^	104.42	1.63 ^b^	4.09 ^a^	150.81
Leaf K	4.50 ^a^	2.66 ^b^	−40.88	4.22 ^a^	1.69 ^b^	−59.94	3.98 ^a^	2.03 ^b^	−49.13
Root K	3.49 ^a^	2.38 ^b^	−31.85	3.32 ^a^	1.42 ^b^	−57.20	3.33 ^a^	1.55 ^b^	−53.33
Leaf K/Na	3.86 ^a^	1.17 ^b^	−69.66	3.05 ^a^	0.48 ^b^	−84.28	3.02 ^a^	0.58 ^b^	−80.66
Root K/Na	2.22 ^a^	0.64 ^b^	−71.07	1.66 ^a^	0.35 ^b^	−78.64	2.09 ^a^	0.40 ^b^	−81.04

Proline (µmol g^−1^ FW), total soluble sugars (TSS, mg g^−1^ FW), relative water content (RWC, %), malondialdehyde (MDA, nM g^−1^ FW), DPPH free radical scavenging (IC50, µg mL^−1^), chlorophyll a (Chla, mg g^−1^ FW), chlorophyll b (Chlb, mg g^−1^ FW), total chlorophyll (Total chl, mg g^−1^ FW), carotenoid (Car, mg g^−1^ FW), Na (mM g^−1^ DW) and K (mM g^−1^ DW) contents and K/Na ratio. Different lowercase letters (a and b) between two treatments for each genotypic group indicate a significant difference between the control and salt stress for a specific trait (LSD test: *p* ≤ 0.05).

**Table 6:** Results of analysis of variance for the cytosine methylation (5-mC) content and expression of the salt-tolerant related genes under the control and salt-stress (250 mM NaCl) treatments.

Source of Variation	df	Mean Square			
		5-mC	*HKT1;5*	*NHX1*	*SOS1*
CS background					
Salt stress (S)	1	442.75 **	13.00 **	91.66 **	6.11 **
Genotype (G)	7	79.60 **	0.23 **	2.41 **	0.27 **
Tissue (T)	1	3703.16 **	9.72 **	23.21 **	0.65 **
S × G	7	1.46 ^ns^	0.06 **	1.67 **	0.08 **
S × T	1	31.61 *	7.09 **	24.01 **	0.15 **
G × T	7	15.58 *	0.09 **	0.37 **	0.01 ^ns^
S × G × T	7	1.15 ^ns^	0.03 *	0.38 **	0.005 ^ns^
Residual	64	6.73	0.01	0.02	0.02
CV (%)		9.63	15.08	9.85	17.81
R background					
Salt stress (S)	1	481.19 **	14.55 **	113.58 **	8.41 **
Genotype (G)	7	51.17 **	0.10 **	2.03 **	0.31 **
Tissue (T)	1	3303.29 **	17.47 **	28.99 **	0.49 **
S × G	7	2.60 ^ns^	0.09 **	1.45 **	0.16 **
S × T	1	54.58 **	7.77 **	32.50 **	0.20 **
G × T	7	14.02 *	0.06 *	0.61 **	0.01 ^ns^
S × G × T	7	2.12 ^ns^	0.05 ^ns^	0.49 **	0.005 ^ns^
Residual	64	6.39	0.02	0.03	0.02
CV (%)		9.01	18.66	10.67	20.11

The symbols along the rows and columns indicate: ^ns^ Non-significant; * *p* ≤ 0.05, ** *p* ≤ 0.01.

## Data Availability

The authors declare that all relevant data are available within the article and its Supplementary Materials.

## Supplementary Materials

The following supporting information can be downloaded at: https://www.mdpi.com/article/10.3390/plants13192673/s1, Figure S1: Mean comparison of proline content, “Chinese spring” (CS), “Roshan” (R) cultivars, *Ae. cylindrica* (AC), and their derived BC_4_F_2_ (tolerant and sensitive) lines; Figure S2: Mean comparison of total soluble sugars (TSS) content, “Chinese spring” (CS), “Roshan” (R) cultivars, *Ae. cylindrica* (AC), and their derived selected lines from backcrosses; Figure S3: Mean comparison of leaf relative water content (RWC), “Chinese spring” (CS), “Roshan” (R) cultivars, Ae. cylindrica and their derived selected lines from backcrosses; Figure S4: Mean comparison of lipid peroxidation (MDA) content, “Chinese spring” (CS), “Roshan” (R) cultivars, *Ae. cylindrica* and their derived selected lines from backcrosses; Figure S5: Mean comparison of chlorophyll content, “Chinese spring” (CS), “Roshan” (R) cultivars, Ae. cylindrica and their derived selected lines from backcrosses; Figure S6: Mean comparison of carotenoid (Car) content, “Chinese spring” (CS), “Roshan” (R) cultivars, *Ae. cylindrica*, and their derived selected lines from backcrosses; Table S1: The average yield and yield components in “Chinese Spring” (CS) derived BC_4_F_2_ lines under normal and salt stress conditions over two crop years; Table S2: The average yield and its related components in “Roshan” (R) derived BC_4_F_2_ lines under normal and salt stress conditions over two crop years; Table S3: The means of the agronomic trait of two genetic backgrounds of wheat (CS and R) under control (0 mM NaCl) and stress (250 mM NaCl) conditions.

## Abbreviations

Ac, *Aegilops cylindrica*; Chla, Chlorophyll a; Chlb, Chlorophyll b; CS, “Chinese Spring” wheat cultivar; DPPH, 2,2-diphenyl-1-picrylhydrazyl; GMP, Geometric mean productivity; GpS, Number of grains per spike; GW, 100-grain weight; HCPC, Hierarchical principal component clustering; HKT, High-affinity potassium transporters; HM, Harmonic mean; MDA, Malondialdehyde; 5-mC, 5-methylcytosine; MP, Mean productivity; *NHX1*, Tonoplast-localized Na^+^/H^+^ antiporter; P5CS, Osmoprotectant pyrroline-5-carboxylate synthetase; PH, Plant height; R, “Roshan” wheat cultivar; ROS, Reactive oxygen species; RWC, Relative water content; SL, Spike length; *SOS1*, Salt-overly sensitive 1; SpP, Number of spikes per plant; SSI, Stress susceptibility index; STI, Stress tolerance index; TBA, Thiobarbituric acid; TCA, Thiobarbituric acid; TOL, Tolerance index; Total Chl, Total chlorophyll; TSS, Total soluble sugars; Yield, Grain yield per plant; YI, Yield index; YSI, Yield stability index.

## References

[1] Arzani A., Ashraf M. (2016). Smart engineering of genetic resources for enhanced salinity tolerance in crop plants. Crit. Rev. Plant Sci..

[2] Homaee M., Feddes R., Dirksen C. (2002). A macroscopic water extraction model for nonuniform transient salinity and water stress. Soil Sci. Soc. Am. J..

[3] Munns R., Gilliham M. (2015). Salinity tolerance of crops–what is the cost?. New Phytol..

[4] Zhu M., Zhou M., Shabala L., Shabala S. (2014). Linking osmotic adjustment and stomatal characteristics with salinity stress tolerance in contrasting barley accessions. Funct. Plant Biol..

[5] Hussain S., Ulhassan Z., Brestic M., Zivcak M., Zhou W., Allakhverdiev S.I., Yang X., Safdar M.E., Yang W., Liu W. (2021). Photosynthesis research under climate change. Photosynth. Res..

[6] Francois L., Maas E., Donovan T., Youngs V. (1986). Effect of salinity on grain yield and quality, vegetative growth, and germination of semi-dwarf and durum wheat. Agron. J..

[7] Arabbeigi M., Arzani A., Majidi M.M. (2019). Expression profiles of *P5CS* and *DREB2* genes under salt stress in *Aegilops cylindrica*. Russ. J. Plant Physiol..

[8] Huang S., Spielmeyer W., Lagudah E.S., Munns R. (2008). Comparative mapping of *HKT* genes in wheat, barley, and rice, key determinants of Na^+^ transport, and salt tolerance. J. Exp. Bot..

[9] Kiani R., Arzani A., Mirmohammady Maibody S. (2021). Polyphenols, flavonoids, and antioxidant activity involved in salt tolerance in wheat, *Aegilops cylindrica* and their amphidiploids. Front. Plant Sci..

[10] Arzani A., Ashraf M. (2017). Cultivated ancient wheats (*Triticum* spp.): A potential source of health-beneficial food products. Compr. Rev. Food Sci. Food Saf..

[11] Kiani R., Arzani A., Mirmohammady Maibody S., Rahimmalek M., Razavi K. (2021). Morpho-physiological and gene expression responses of wheat by *Aegilops cylindrica* amphidiploids to salt stress. Plant Cell Tissue Organ Cult..

[12] Singh P.K., Gautam S. (2013). Role of salicylic acid on physiological and biochemical mechanism of salinity stress tolerance in plants. Acta Physiol. Plant..

[13] Soundararajan P., Manivannan A., Jeong B.R. (2019). Different antioxidant defense systems in halophytes and glycophytes to overcome salinity stress. Sabkha Ecosystems: Volume VI: Asia/Pacific.

[14] Liang W., Ma X., Wan P., Liu L. (2018). Plant salt-tolerance mechanism: A review. Biochem. Biophys. Res. Commun..

[15] Darko E., Khalil R., Dobi Z., Kovács V., Szalai G., Janda T., Molnár I. (2020). Addition of *Aegilops biuncialis* chromosomes 2M or 3M improves the salt tolerance of wheat in different way. Sci. Rep..

[16] Kiani R., Arzani A., Habibi F. (2015). Physiology of salinity tolerance in *Aegilops cylindrica*. Acta Physiol. Plant..

[17] Pan T., Liu M., Kreslavski V.D., Zharmukhamedov S.K., Nie C., Yu M., Kuznetsov V.V., Allakhverdiev S.I., Shabala S. (2021). Non-stomatal limitation of photosynthesis by soil salinity. Crit. Rev. Environ. Sci. Technol..

[18] Shabala S. (2017). Signalling by potassium: Another second messenger to add to the list?. J. Exp. Bot..

[19] Wu H., Zhang X., Giraldo J.P., Shabala S. (2018). It is not all about sodium: Revealing tissue specificity and signalling roles of potassium in plant responses to salt stress. Plant Soil.

[20] Duan H.-R., Ma Q., Zhang J.-L., Hu J., Bao A.-K., Wei L., Wang Q., Luan S., Wang S.-M. (2015). The inward-rectifying K^+^ channel SsAKT1 is a candidate involved in K^+^ uptake in the halophyte Suaeda salsa under saline condition. Plant Soil.

[21] Yan K., Shao H., Shao C., Chen P., Zhao S., Brestic M., Chen X. (2013). Physiological adaptive mechanisms of plants grown in saline soil and implications for sustainable saline agriculture in coastal zone. Acta Physiol. Plant..

[22] Ebrahim F., Arzani A., Rahimmalek M., Sun D., Peng J. (2020). Salinity tolerance of wild barley *Hordeum vulgare* ssp. spontaneum. Plant Breed..

[23] Rubio F., Nieves-Cordones M., Horie T., Shabala S. (2020). Doing ‘business as usual’comes with a cost: Evaluating energy cost of maintaining plant intracellular K^+^ homeostasis under saline conditions. New Phytol..

[24] Arabbeigi M., Arzani A., Majidi M.M., Kiani R., Sayed Tabatabaei B.E., Habibi F. (2014). Salinity tolerance of *Aegilops cylindrica* genotypes collected from hyper-saline shores of Uremia Salt Lake using physiological traits and SSR markers. Acta Physiol. Plant..

[25] Ariño-Estrada G., Mitchell G.S., Saha P., Arzani A., Cherry S.R., Blumwald E., Kyme A.Z. (2019). Imaging salt uptake dynamiCS in plants using PET. Sci. Rep..

[26] Arabbeigi M., Arzani A., Majidi M.M., Sayed-Tabatabaei B.E., Saha P. (2018). Expression pattern of salt tolerance-related genes in *Aegilops cylindrica*. Physiol. Mol. Biol. Plants.

[27] Abdulraheem M.I., Xiong Y., Moshood A.Y., Cadenas-Pliego G., Zhang H., Hu J. (2024). Mechanisms of plant epigenetic regulation in response to plant stress: Recent discoveries and implications. Plants.

[28] Hoseini M., Arzani A. (2023). Epigenetic adaptation to drought and salinity in crop plants. J. Plant Mol. Breed..

[29] Kumar S., Seem K., Kumar S., Singh A., Krishnan S.G., Mohapatra T. (2024). DNA methylome analysis provides insights into gene regulatory mechanism for better performance of rice under fluctuating environmental conditions: Epigenomics of adaptive plasticity. Planta.

[30] Li B., Cai H., Liu K., An B., Wang R., Yang F., Zeng Z., Jiao C., Xu Y. (2023). DNA methylation alterations and their association with high temperature tolerance in rice anthesis. J. Plant Growth Regul..

[31] Şahin Z., Ağar G., Yiğider E., Aydın M. (2024). Effects of Selenium on DNA Methylation and Genomic Instability Induced by Drought Stress in Wheat (*Triticum aestivum* L.). Türkiye Tarımsal Araştırmalar Derg..

[32] Stadnik B., Tobiasz-Salach R., Mazurek M. (2022). Effect of silicon on oat salinity tolerance: Analysis of the epigenetic and physiological response of plants. Agriculture.

[33] Tobiasz-Salach R., Mazurek M., Jacek B. (2023). Physiological, biochemical, and epigenetic reaction of maize (*Zea mays* L.) to cultivation in conditions of varying soil salinity and foliar application of silicon. Int. J. Mol. Sci..

[34] Jiabu D., Yu M., Xu Q., Yang H., Mu W., Basang Y. (2023). Genome-wide DNA methylation dynamics during drought responsiveness in Tibetan hulless barley. J. Plant Growth Regul..

[35] Naderi S., Maali-Amiri R., Sadeghi L., Hamidi A. (2024). Physio-biochemical and DNA methylation analysis of the defense response network of wheat to drought stress. Plant Physiol. Biochem..

[36] Kiani R., Arzani A., Maibody S., Rahimmalek M., Ayers T. (2021). Hybridization of wheat and *Aegilops cylindrica*: Development, karyomorphology, DNA barcoding and salt tolerance of the amphidiploids. J. Plant Biochem. Biotechnol..

[37] Fernandez G.C.J., Kuo C.G. (1993). Effective selection criteria for assessing plant stress tolerance. Adaptation of Food Crops to Temperature and Water Stress.

[38] Rosielle A., Hamblin J. (1981). Theoretical aspects of selection for yield in stress and non-stress environment 1. Crop Sci..

[39] Fischer R.A., Maurer R. (1978). Drought resistance in spring wheat cultivars. I. Grain yield responses. Aust. J. Agric. Res..

[40] Bouslama M., Schapaugh W.T. (1984). Stress tolerance in soybeans. I. Evaluation of three screening techniques for heat and drought tolerance 1. Crop Sci..

[41] Gavuzzi P., Rizza F., Palumbo M., Campanile R.G., Ricciardi G.L., Borghi B. (1997). Evaluation of field and laboratory predictors of drought and heat tolerance in winter cereals. Can. J. Plant Sci..

[42] Schneider K.A., Rosales-Serna R., Ibarra-Perez F., Cazares-Enriquez B., Acosta-Gallegos J.A., Ramirez-Vallejo P., Wassimi N., Kelly J.D. (1997). Improving common bean performance under drought stress. Crop Sci..

[43] Hossain A.B.S., Sears A.G., Cox T.S., Paulsen G.M. (1990). Desiccation tolerance and its relationship to assimilate partitioning in winter wheat. Crop Sci..

[44] Bates L.S., Waldren R., Teare I. (1973). Rapid determination of free proline for water-stress studies. Plant Soil.

[45] Irigoyen J., Einerich D., Sánchez-Díaz M. (1992). Water stress induced changes in concentrations of proline and total soluble sugars in nodulated alfalfa (*Medicago sativa*) plants. Physiol. Plant..

[46] Taulavuori E., Hellström E.K., Taulavuori K., Laine K. (2001). Comparison of two methods used to analyse lipid peroxidation from *Vaccinium myrtillus* (L.) during snow removal, reacclimation and cold acclimation. J. Exp. Bot..

[47] Sharma O.P., Bhat T.K. (2009). DPPH antioxidant assay revisited. Food Chem..

[48] Lichtenthaler H.K., Buschmann C. (2001). Chlorophylls and carotenoids: Measurement and characterization by UV-VIS spectroscopy. Curr. Protoc. Food Anal. Chem..

[49] Houshmand S., Arzani A., Maibody SA M., Feizi M. (2005). Evaluation of salt-tolerant genotypes of durum wheat derived from in vitro and field experiments. Field Crops Res..

[50] Kump B., Javornik B. (1996). Evaluation of genetic variability among common buckwheat (*Fagopyrum esculentum* Moench) populations by RAPD markers. Plant Sci..

[51] Murray M.G., Thompson W.F. (1980). Rapid isolation of high-molecular-weight plant DNA. Nucleic Acids Res..

[52] Livak K.J., Schmittgen T.D. (2001). Analysis of relative gene expression data using real-time quantitative PCR and the 2^−ΔΔCT^ method. Methods.

[53] Arzani A. (2008). Improving salinity tolerance in crop plants: A biotechnological view. In Vitro Cell. Dev. Biol.-Plant.

[54] Munns R., Tester M. (2008). Mechanisms of salinity tolerance. Annu. Rev. Plant Biol..

[55] Henry R.J., Nevo E. (2014). Exploring natural selection to guide breeding for agriculture. Plant Biotechnol. J..

[56] Zeibig F., Kilian B., Frei M. (2022). The grain quality of wheat wild relatives in the evolutionary context. Theor. Appl. Genet..

[57] Gharaghanipor N., Arzani A., Rahimmalek M., Ravash R. (2022). Physiological and transcriptome indicators of salt tolerance in wild and cultivated barley. Front. Plant Sci..

[58] Kumar S., Beena A., Awana M., Singh A. (2017). Physiological, biochemical, epigenetic and molecular analyses of wheat (*Triticum aestivum*) genotypes with contrasting salt tolerance. Front. Plant Sci..

[59] Romero-Aranda M.R., Jurado O., Cuartero J. (2006). Silicon alleviates the deleterious salt effect on tomato plant growth by improving plant water status. J. Plant Physiol..

[60] Radi A.A., Farghaly F.A., Hamada A.M. (2013). Physiological and biochemical responses of salt-tolerant and salt-sensitive wheat and bean cultivars to salinity. J. Biol. Earth Sci..

[61] Zeeshan M., Lu M., Sehar S., Holford P., Wu F. (2020). Comparison of biochemical, anatomical, morphological, and physiological responses to salinity stress in wheat and barley genotypes deferring in salinity tolerance. Agronomy.

[62] Ehtaiwesh A., Sunoj V.S.J., Djanaguiraman M., Prasad P.V.V. (2024). Response of winter wheat genotypes to salinity stress under controlled environments. Front. Plant Sci..

[63] Arzani A. (2018). Manipulating programmed cell death pathways for enhancing salinity tolerance in crops. Salinity Responses and Tolerance in Plants, Volume 2: Exploring RNAi, Genome Editing and Systems Biology.

[64] Kaur G., Asthir B., Bains N. (2018). Modulation of proline metabolism under drought and salt stress conditions in wheat seedlings. Indian J. Biochem. Biophys.

[65] Matković Stojšin M., Petrović S., Banjac B., Zečević V., Roljević Nikolić S., Majstorović H., Đorđević R., Knežević D. (2022). Assessment of genotype stress tolerance as an effective way to sustain wheat production under salinity stress conditions. Sustainability.

[66] Rodrigues M.J., Monteiro I., Castañeda-Loaiza V., Placines C., Oliveira M.C., Reis C., Caperta A.D., Soares F., Pousão-Ferreira P., Pereira C. (2020). Growth performance, in vitro antioxidant properties and chemical composition of the halophyte *Limonium algarvense* Erben are strongly influenced by the irrigation salinity. Ind. Crops Prod..

[67] Sadak M.S., Talaat I.M. (2021). Attenuation of negative effects of saline stress in wheat plant by chitosan and calcium carbonate. Bull. Natl. Res. Cent..

[68] Stojšin M.M., Petrović S., Dimitrijević M., Malenčić D., Zečević V., Banjac B., Knežević D. (2022). Effect of salinity stress on antioxidant activity and grain yield of different wheat genotypes. Turk. J. Field Crops.

[69] Saddiq M.S., Iqbal S., Hafeez M.B., Ibrahim A.M., Raza A., Fatima E.M., Baloch H., Jahanzaib Woodrow P., Ciarmiello L.F. (2021). Effect of salinity stress on physiological changes in winter and spring wheat. Agronomy.

[70] Wu H., Shabala L., Zhou M., Su N., Wu Q., Ul-Haq T., Zhu J., Mancuso S., Azzarello E., Shabala S. (2019). Root vacuolar Na^+^ sequestration but not exclusion from uptake correlates with barley salt tolerance. Plant J..

[71] van Bezouw R.F.H.M., Janssen E.M., Ashrafuzzaman M., Ghahramanzadeh R., Kilian B., Graner A., Visser R.G.F., van der Linden C.G. (2019). Shoot sodium exclusion in salt stressed barley (*Hordeum vulgare* L.) is determined by allele specific increased expression of *HKT1;5*. J. Plant Physiol..

[72] Byrt C.S., Xu B., Krishnan M., Lightfoot D.J., Athman A., Jacobs A.K., Watson-Haigh N.S., Plett D., Munns R., Tester M. (2014). The Na^+^ transporter, Ta hkt 1; 5-d, limits shoot Na^+^ accumulation in bread wheat. Plant J..

[73] Oyiga B.C., Sharma R.C., Baum M., Ogbonnaya F.C., Léon J., Ballvora A. (2018). Allelic variations and differential expressions detected at quantitative trait loci for salt stress tolerance in wheat. Plant Cell Environ..

[74] Rus A., Lee B.-H., Munoz-Mayor A., Sharkhuu A., Miura K., Zhu J.-K., Bressan R.A., Hasegawa P.M. (2004). AtHKT1 facilitates Na^+^ homeostasis and K^+^ nutrition in planta. Plant Physiol..

[75] Venkataraman G., Shabala S., Véry A.A., Hariharan G.N., Somasundaram S., Pulipati S., Sellamuthu G., Harikrishnan M., Kumkum K., Shabala L. (2021). To exclude or to accumulate? Revealing the role of the sodium HKT1;5 transporter in plant adaptive responses to varying soil salinity. Plant Physiol. Biochem..

[76] Nawaz I., Iqbal M., Hakvoort H.W., Bliek M., de Boer B., Schat H. (2014). Expression levels and promoter activities of candidate salt tolerance genes in halophytic and glycophytic Brassicaceae. Environ. Exp. Bot..

[77] Xu B., Hrmova M., Gilliham M. (2020). High affinity Na^+^ transport by wheat HKT1;5 is blocked by K^+^. Plant Direct.

[78] Arzani A., Kumar S., Mansour M.M.F. (2023). Salt tolerance in plants: Molecular and functional adaptations. Front. Plant Sci..

[79] Ashapkin V.V., Kutueva L.I., Aleksandrushkina N.I., Vanyushin B.F. (2020). Epigenetic mechanisms of plant adaptation to biotic and abiotic stresses. Int. J. Mol. Sci..

[80] Steward N., Ito M., Yamaguchi Y., Koizumi N., Sano H. (2002). Periodic DNA methylation in maize nucleosomes and demethylation by environmental stress. J. Biol. Chem..

