# Supplementary material for: Agro-Physiological and DNA Methylation Responses to Salinity Stress in Wheat (Triticum aestivum L.), Aegilops cylindrica Host, and Their Introgressed Lines
Source: Plants (Basel). 2024 Sep 24;13(19):2673. doi: 10.3390/plants13192673 (PMC11479238; doi:10.3390/plants13192673)
Supplement: Supplementary file 1 [file plants-13-02673-s001.zip › plants-3189451-supplementary.pdf]

Table S1: The average yield and yield components in 'Chinese Spring' (CS) derived BC4F2 lines under normal and salt stress conditions over two crop years.

No.	Line	Grain yield	Grain weight	Grains per spike	Spikes per plant	Spike length	Plant height
**Control (0 mM NaCl)**							
1	CS	2.29	1.97	31.87	3.72	8.13	88.35
2	C1	1.10	2.20	22.32	2.28	8.23	76.31
3	C2	1.22	1.98	30.36	2.06	7.30	78.65
4	C3	2.45	2.72	28.66	3.17	7.96	81.24
5	C4	2.04	2.28	24.67	3.87	8.24	83.62
6	C5	1.79	2.12	22.85	3.78	7.76	84.75
7	C6	1.87	1.83	26.44	4.02	7.71	88.48
8	C7	3.33	2.43	36.24	4.00	7.93	78.06
9	C8	2.26	2.20	27.40	3.81	8.88	90.01
10	C9	2.90	2.16	34.08	4.22	7.68	92.89
11	C10	1.42	2.26	29.05	2.14	8.37	77.69
12	C11	1.74	2.27	39.71	2.01	8.22	80.70
13	C12	1.78	2.71	27.85	2.33	8.18	73.20
14	C13	2.22	3.29	24.04	2.82	7.89	83.28
15	C14	1.15	2.86	27.45	1.47	8.11	81.54
16	C16	3.85	2.60	32.63	9.42	8.33	87.00
17	C17	1.67	2.26	25.23	11.85	7.65	87.10
18	C18	1.93	1.68	28.87	4.30	8.77	83.51
19	C19	1.15	2.15	24.02	2.27	7.80	81.18
20	C20	2.08	2.18	35.62	6.13	7.89	72.74
21	C21	3.51	2.36	36.48	4.14	8.56	83.57
22	C22	0.99	1.88	24.80	2.11	8.78	79.03
23	C23	1.26	1.99	23.72	4.43	7.19	77.00
24	C24	2.00	2.14	15.82	7.39	8.70	76.99
25	C25	0.92	2.21	25.53	1.64	8.07	83.01
26	C26	1.79	2.16	25.26	3.31	8.29	80.81
27	C27	2.30	1.98	30.98	3.85	8.59	81.54
28	C28	1.90	3.49	27.85	2.01	8.32	83.96
29	C29	2.88	3.04	22.40	4.29	8.35	82.22
30	C30	2.66	2.14	21.27	6.02	7.90	78.66
31	C31	1.59	2.10	26.26	3.33	7.56	84.12
32	C33	1.41	2.06	17.03	4.12	8.74	89.67
33	C34	1.94	2.82	28.02	2.50	8.04	88.02
34	C35	1.33	2.81	20.13	2.33	7.99	80.19
35	C36	2.48	3.06	27.56	2.98	8.36	90.21
36	C37	2.53	2.16	32.04	3.71	7.48	81.03
37	C38	1.19	2.08	25.88	2.21	8.46	82.24
38	C39	1.91	3.05	24.24	2.57	7.86	86.41
39	C40	1.58	2.23	24.86	2.90	7.32	86.75
40	C41	1.25	2.92	24.39	1.80	8.09	90.03

41	C42	1.48	2.17	34.07	2.04	7.33	69.87
42	C43	1.26	2.31	25.33	2.17	8.44	85.81
43	C44	1.21	1.97	20.25	3.21	9.01	85.22
44	C45	1.33	2.28	20.71	2.89	7.96	73.71
45	C46	2.71	2.01	22.97	8.42	7.20	82.55
46	C47	1.81	2.27	21.95	3.69	8.16	79.65
47	C48	0.85	2.04	17.04	2.51	7.55	79.69
48	C49	2.16	2.97	27.65	8.47	7.98	80.15
49	C50	2.41	2.07	21.08	5.80	8.57	86.61
50	C51	1.01	3.10	22.96	1.41	9.95	74.64
51	C52	2.21	2.68	25.19	3.74	8.03	82.72
52	C53	1.39	1.82	18.44	4.43	8.08	70.84
53	C54	1.08	2.74	22.24	1.78	7.62	71.77
54	C55	1.73	2.94	29.56	1.99	7.88	85.92
55	C56	1.32	2.59	28.47	1.82	7.97	85.25
56	C57	1.77	2.04	30.67	2.91	7.69	74.99
57	C58	0.95	2.09	25.21	1.88	8.08	80.73
58	C59	1.78	2.20	22.87	3.72	7.96	79.83
59	C60	1.20	2.55	25.65	1.85	7.56	79.37
60	C61	1.94	2.79	27.66	2.52	8.20	78.28
61	C62	2.12	2.99	18.73	10.74	7.42	80.98
62	C63	1.62	2.06	21.59	3.71	7.93	82.83
63	C64	2.07	2.37	35.14	2.57	8.39	73.36
64	C65	2.82	4.02	24.22	2.89	7.12	83.22
65	C66	1.71	2.98	29.87	1.95	8.78	78.63
66	C67	1.72	3.01	28.53	2.03	8.46	85.35
67	C68	1.06	1.91	24.02	2.39	7.48	71.24
68	C70	4.02	2.54	31.54	5.28	8.79	81.66
69	C72	1.49	2.10	27.45	2.59	7.90	72.64
70	C73	1.69	2.01	25.99	3.34	8.02	79.51
71	C74	3.07	3.02	33.67	3.08	7.78	84.62
72	C75	2.48	2.30	26.06	4.24	7.97	84.92
73	C80	2.08	2.88	28.83	2.53	8.37	83.25
**Salt stress (250 mM NaCl)**							
1	CS	0.54	1.92	16.37	1.74	7.64	62.24
2	C1	0.40	1.36	14.64	2.22	7.31	58.97
3	C2	0.71	1.75	18.79	2.33	6.62	61.74
4	C3	1.42	2.53	22.45	2.56	7.84	71.68
5	C4	0.76	2.01	21.89	1.75	7.10	65.78
6	C5	0.79	1.83	15.11	3.03	6.93	64.66
7	C6	1.50	1.53	20.54	5.00	7.74	62.05
8	C7	1.08	1.71	16.98	4.10	8.01	63.41
9	C8	1.14	1.79	24.81	2.71	8.31	67.93
10	C9	0.88	1.62	20.44	2.71	7.66	61.94
11	C10	1.27	1.46	15.80	6.17	6.86	59.01
12	C11	0.76	1.55	20.71	2.56	7.21	63.74

13	C12	0.58	1.69	21.31	1.67	7.13	63.08
14	C13	0.87	1.86	16.02	3.01	7.24	66.68
15	C14	0.96	1.91	25.96	1.99	7.28	63.16
16	C16	1.07	1.78	17.17	3.82	8.09	66.02
17	C17	0.63	1.34	7.66	5.92	6.80	55.45
18	C18	0.67	1.61	17.41	2.62	8.05	67.25
19	C19	1.01	1.71	20.72	3.62	7.46	67.85
20	C20	1.06	1.55	15.32	4.60	7.45	60.73
21	C21	0.52	1.88	20.91	1.38	7.67	61.10
22	C22	0.65	1.65	21.99	1.76	8.04	67.11
23	C23	0.47	1.50	16.32	1.85	6.75	58.56
24	C24	0.60	1.77	13.36	2.52	7.59	63.78
25	C25	0.87	2.27	22.60	1.72	7.82	63.48
26	C26	0.80	1.68	19.09	2.69	7.86	60.38
27	C27	0.89	2.04	25.31	1.77	9.27	65.11
28	C28	1.26	3.39	16.14	2.39	7.82	55.57
29	C29	1.41	1.74	18.63	4.73	7.57	64.76
30	C30	0.54	1.63	15.58	2.21	7.76	60.69
31	C31	0.69	1.76	19.45	2.05	7.53	53.11
32	C33	1.24	1.81	15.07	4.68	8.72	71.46
33	C34	0.53	1.80	17.38	1.65	6.86	60.42
34	C35	0.83	1.81	16.99	2.83	7.31	66.98
35	C36	2.16	1.90	23.90	4.96	7.43	64.11
36	C37	0.79	1.79	22.22	1.96	6.75	64.07
37	C38	0.49	1.43	13.38	2.63	7.32	63.75
38	C39	1.28	1.91	17.81	4.37	6.96	60.64
39	C40	1.27	2.16	22.60	2.75	7.40	67.47
40	C41	0.71	1.46	14.52	3.54	7.94	62.53
41	C42	1.37	1.82	25.82	3.07	7.15	64.80
42	C43	0.64	2.13	22.75	1.32	8.88	69.03
43	C44	0.94	1.28	16.77	5.80	7.25	60.46
44	C45	1.12	2.12	19.04	3.12	8.01	64.77
45	C46	0.92	1.66	15.71	3.73	7.37	64.72
46	C47	1.03	1.83	19.66	2.99	7.91	70.48
47	C48	0.61	1.44	11.38	4.02	7.00	56.98
48	C49	0.53	1.47	11.53	3.49	7.45	60.02
49	C50	0.83	1.99	18.42	2.30	7.41	61.36
50	C51	0.93	1.92	20.62	2.60	8.30	65.01
51	C52	1.85	1.97	22.13	4.21	7.48	67.31
52	C53	1.10	1.56	16.78	4.80	7.55	54.26
53	C54	0.48	1.36	13.96	2.83	7.03	51.75
54	C55	1.20	1.47	12.86	7.43	6.31	60.71
55	C56	1.14	1.81	19.55	3.94	7.50	63.05
56	C57	0.56	2.03	24.44	1.22	7.64	68.90
57	C58	0.87	1.83	17.69	3.04	7.37	65.66
58	C59	1.50	1.51	20.25	6.05	8.17	62.68

59	C60	0.88	1.53	15.55	4.66	7.40	60.11
60	C61	0.41	1.65	14.47	1.75	7.14	56.54
61	C62	0.48	1.25	10.89	4.31	6.18	49.20
62	C63	0.80	2.12	19.85	1.97	6.92	67.36
63	C64	0.91	1.93	17.42	2.83	8.14	62.83
64	C65	1.65	2.28	21.69	3.44	6.78	68.04
65	C66	1.48	2.15	26.88	2.79	9.09	67.93
66	C67	1.06	1.92	21.61	2.60	7.34	62.83
67	C68	0.28	1.23	15.31	1.45	6.65	57.84
68	C70	1.52	1.97	17.42	5.83	8.21	65.57
69	C72	1.39	1.86	20.92	3.92	7.69	68.50
70	C73	1.09	1.79	22.60	3.05	6.94	66.33
71	C74	1.30	1.80	21.21	3.65	7.46	63.48
72	C75	1.02	1.89	17.29	3.41	7.23	63.42
73	C80	1.75	2.04	22.94	3.91	6.99	60.36

Table S2: The average yield and its related components in "Roshan" (R) derived BC4F2 lines under normal and salt stress conditions over two crop years.

No.	Line	Grain yield	Grain weight	Grains per spike	Spikes per plant	Spike length	Plant height
**Control (0 mM NaCl)**							
1	R	3.37	3.55	26.00	3.79	10.26	95.03
2	R1	2.04	3.40	36.50	1.76	10.68	88.24
3	R2	3.30	3.58	34.17	2.83	10.47	85.52
4	R3	2.16	3.74	31.59	2.01	10.29	85.58
5	R4	3.66	4.00	30.73	3.03	11.24	89.84
6	R5	2.88	4.66	28.22	2.35	10.50	86.25
7	R6	3.02	3.00	30.29	3.43	10.69	86.91
8	R7	2.81	3.45	38.26	2.18	11.21	84.87
9	R8	3.01	3.88	42.05	1.88	11.17	89.82
10	R10	3.70	3.60	28.26	3.68	9.83	88.12
11	R12	2.59	3.61	35.53	2.05	10.39	87.00
12	R13	4.45	3.40	32.55	4.11	10.82	96.00
13	R14	2.30	5.11	27.05	1.71	10.92	87.76
14	R15	2.82	4.33	35.99	1.92	11.19	80.46
15	R16	3.56	3.41	40.33	2.65	10.94	88.96
16	R17	2.55	3.66	37.81	1.79	10.37	86.36
17	R18	2.86	3.38	28.83	3.13	10.86	87.42
18	R19	2.90	3.40	43.58	1.99	11.32	91.20
19	R20	5.54	3.22	36.22	5.00	11.08	86.72
20	R21	2.21	3.74	30.59	1.94	10.49	84.50
21	R22	2.35	3.29	44.55	1.62	11.34	82.27
22	R23	3.84	4.69	25.21	3.36	10.88	83.08
23	R24	3.07	6.26	21.13	2.84	10.45	94.44
24	R25	1.79	3.16	29.84	1.88	10.77	84.08
25	R26	2.76	4.23	34.88	1.89	10.35	79.74
26	R27	2.11	3.43	39.29	1.56	9.95	87.92
27	R29	2.60	4.11	33.39	1.85	11.14	87.21
28	R30	3.07	2.80	38.37	2.96	11.63	91.55
29	R31	2.70	2.90	34.60	2.71	10.64	86.25
30	R32	3.02	3.74	29.61	2.81	9.41	89.42
31	R33	2.24	3.08	31.17	2.42	9.96	79.77
32	R34	2.27	4.37	29.99	1.80	10.74	87.33
33	R35	2.50	3.15	32.64	2.54	10.99	82.55
34	R36	2.19	3.50	34.38	1.82	10.57	86.77
35	R37	2.77	3.62	35.18	2.14	10.87	76.21
36	R38	2.41	3.01	35.54	2.25	10.30	85.94
37	R39	2.26	3.30	34.28	1.98	9.83	78.74
38	R40	2.74	3.50	36.72	2.17	11.21	90.44
39	R41	1.78	2.97	31.80	1.85	10.16	80.90
40	R42	3.90	3.37	31.86	3.72	10.80	78.60

41	R43	2.66	4.08	33.41	1.96	10.63	92.12
42	R44	3.19	3.99	30.50	2.68	9.88	88.58
43	R45	3.59	2.92	34.35	3.60	11.15	82.73
44	R46	4.43	3.70	30.16	4.03	10.04	88.44
45	R47	1.82	2.58	29.47	2.38	10.79	86.43
46	R48	1.80	3.41	33.58	1.58	10.40	77.83
47	R49	2.84	2.73	46.38	2.40	10.67	81.39
48	R50	2.49	3.36	33.53	2.27	11.16	84.74
49	R51	3.06	2.91	34.43	3.15	10.62	87.43
50	R52	2.08	2.77	38.17	2.06	10.43	88.08
51	R53	1.87	3.21	28.92	2.06	10.36	81.05
52	R54	2.82	3.97	42.33	1.80	11.63	88.29
53	R55	2.01	3.23	42.90	1.48	10.66	74.71
54	R56	4.51	3.73	31.34	3.95	10.72	84.95
55	R57	2.64	3.49	36.69	2.06	11.34	85.08
56	R58	2.44	2.93	44.00	1.90	11.00	83.06
57	R59	3.21	3.47	40.27	2.31	11.01	85.98
58	R60	3.92	4.98	24.99	3.30	10.79	89.62
59	R61	2.79	3.25	36.72	2.35	11.27	87.59
60	R62	2.96	4.40	20.40	3.81	10.89	82.22
61	R63	4.14	3.20	45.06	2.88	11.56	97.41
62	R64	2.21	3.50	30.67	1.99	10.03	79.26
63	R65	2.94	4.72	33.50	1.96	11.05	83.90
64	R66	3.20	3.31	46.31	2.12	11.50	85.49
65	R67	3.20	3.46	35.50	2.70	10.63	82.25
66	R68	3.08	4.68	35.38	1.92	11.35	86.62
67	R70	2.43	3.42	32.75	2.15	10.48	85.16
68	R71	2.74	2.95	29.92	3.31	10.73	86.17
69	R72	2.55	3.40	32.66	2.29	12.12	90.86
70	R73	2.62	2.91	36.91	2.43	11.15	88.57
71	R74	2.00	3.83	33.50	1.74	10.42	82.92
72	R75	4.90	3.18	43.59	3.64	11.24	93.65
73	R76	3.27	3.78	35.85	2.45	10.43	83.66
74	R77	3.05	3.48	35.38	2.47	10.36	86.93
75	R78	1.99	3.15	31.75	2.00	10.34	80.87
76	R79	3.74	3.24	33.73	3.69	11.06	83.63
77	R80	1.65	2.94	49.31	1.50	11.09	79.18
78	R81	3.95	3.30	35.63	3.40	10.82	86.28
79	R82	2.86	3.65	31.55	2.48	10.73	84.37
80	R83	4.12	3.88	34.56	3.11	11.11	76.70
81	R84	3.71	4.00	33.56	3.16	10.64	86.58
82	R85	2.59	2.58	43.12	2.44	11.07	82.68
83	R86	3.78	3.24	39.10	3.03	10.61	81.43
84	R87	1.90	3.23	29.39	1.89	10.85	80.21
85	R9	2.60	3.83	30.87	2.24	10.31	71.94
**Salt stress (250 mM NaCl)**							

1	R	1.64	3.23	29.10	1.92	9.58	71.36
2	R1	1.90	3.30	24.25	2.48	10.86	74.10
3	R2	1.56	3.31	26.14	2.30	9.69	62.64
4	R3	1.89	3.45	25.92	2.39	9.77	62.72
5	R4	2.28	3.44	25.34	2.84	9.06	73.60
6	R5	0.84	3.52	22.16	1.39	9.36	64.99
7	R6	1.37	2.88	25.50	2.32	9.52	61.58
8	R7	1.22	2.40	30.67	1.74	10.08	62.11
9	R8	1.82	3.52	27.95	1.89	9.60	68.72
10	R10	2.00	3.16	28.91	2.61	9.31	68.02
11	R12	1.90	3.39	28.00	2.28	10.14	64.44
12	R13	2.29	2.74	23.59	3.58	9.07	61.91
13	R14	1.58	4.10	23.31	1.75	10.15	67.83
14	R15	1.62	3.10	34.43	1.49	9.63	63.77
15	R16	2.06	2.80	26.32	2.96	10.27	69.84
16	R17	1.12	3.07	27.66	1.36	9.26	63.78
17	R18	2.25	3.38	30.64	2.27	10.79	66.84
18	R19	1.79	2.91	27.54	2.30	9.98	74.42
19	R20	1.44	2.95	27.68	1.97	8.83	67.71
20	R21	1.28	3.29	26.83	1.77	10.03	63.89
21	R22	1.85	2.89	30.42	2.41	10.67	70.14
22	R23	2.00	3.48	32.29	1.83	10.15	65.73
23	R24	1.45	3.61	25.75	1.79	8.84	66.52
24	R25	1.54	3.18	25.70	2.02	10.81	68.04
25	R26	1.91	3.10	28.53	2.61	10.95	68.78
26	R27	1.79	3.53	27.08	2.06	10.71	65.97
27	R29	1.30	3.59	23.37	1.69	9.74	68.03
28	R30	1.22	2.05	23.87	2.47	9.71	61.82
29	R31	1.34	3.19	21.80	2.23	9.74	59.94
30	R32	1.68	3.03	25.98	2.19	9.49	70.34
31	R33	1.09	2.97	21.19	2.37	8.99	66.23
32	R34	1.60	3.42	25.60	1.95	10.01	66.94
33	R35	1.73	2.27	26.78	3.22	10.57	68.01
34	R36	1.54	3.19	26.61	1.83	10.19	67.48
35	R37	2.50	2.98	29.92	3.11	10.83	69.34
36	R38	1.38	3.89	28.97	1.20	9.42	62.57
37	R39	2.14	2.67	26.66	3.19	10.10	66.75
38	R40	1.64	3.19	23.29	2.28	9.63	65.00
39	R41	1.68	3.52	25.35	2.05	9.88	62.53
40	R42	2.14	3.70	28.71	2.21	9.52	71.63
41	R43	1.78	3.59	31.17	1.86	9.69	67.77
42	R44	1.88	3.10	27.23	2.49	9.81	68.11
43	R45	1.29	3.60	23.24	1.64	9.19	59.95
44	R46	1.30	3.31	22.07	1.86	9.14	61.06
45	R47	0.93	3.05	26.50	1.26	9.14	65.35
46	R48	1.20	3.14	20.67	2.08	9.93	60.89

47	R49	0.91	3.52	24.85	1.19	9.15	62.35
48	R50	2.28	3.41	25.97	3.01	11.29	67.34
49	R51	1.16	3.41	23.37	1.56	9.14	61.36
50	R52	1.68	3.10	24.28	2.32	10.79	63.48
51	R53	1.35	3.05	20.84	2.35	10.80	68.22
52	R54	1.32	3.37	24.15	1.72	9.14	66.74
53	R55	1.12	3.48	24.38	1.33	9.96	66.28
54	R56	2.05	2.39	25.26	3.57	9.25	68.77
55	R57	1.08	3.81	24.76	1.15	9.24	57.03
56	R58	1.85	2.94	21.47	3.11	9.69	64.02
57	R59	1.83	3.37	31.38	1.78	9.18	71.68
58	R60	1.78	3.44	25.01	2.57	9.65	65.76
59	R61	1.42	2.66	27.25	2.30	10.07	68.03
60	R62	1.48	3.27	23.63	2.17	9.25	67.97
61	R63	1.63	2.81	26.53	2.64	9.50	67.67
62	R64	1.62	3.22	25.73	2.22	10.12	69.99
63	R65	2.12	3.40	26.57	2.50	11.01	77.55
64	R66	1.35	3.07	24.15	1.93	9.17	59.49
65	R67	1.29	3.11	26.93	1.71	9.74	62.83
66	R68	1.40	2.98	19.69	2.44	9.91	63.16
67	R70	1.22	3.59	22.97	1.45	8.89	65.33
68	R71	1.17	3.24	25.76	1.43	9.08	60.32
69	R72	0.87	3.91	18.14	1.40	9.64	62.27
70	R73	1.50	3.15	26.34	1.75	9.40	65.75
71	R74	1.01	3.06	23.27	1.95	9.80	62.78
72	R75	1.85	3.65	28.08	2.06	9.52	65.54
73	R76	1.12	3.80	23.93	1.22	8.57	56.86
74	R77	2.39	3.46	31.26	2.30	10.86	71.67
75	R78	1.60	2.95	25.42	2.23	10.26	67.26
76	R79	1.01	3.32	23.19	1.36	9.47	60.45
77	R80	0.43	3.86	21.29	0.61	9.91	51.01
78	R81	0.87	3.35	24.83	1.10	9.51	58.62
79	R82	1.62	3.63	23.26	1.99	9.40	67.80
80	R83	2.10	2.91	30.90	2.47	10.71	73.54
81	R84	1.79	3.30	28.94	2.14	10.08	66.22
82	R85	1.19	3.34	27.74	1.45	9.19	64.00
83	R86	3.09	3.82	28.18	3.05	10.89	66.42
84	R87	1.19	2.99	23.40	1.74	9.29	60.18
85	R9	1.24	3.05	25.50	1.70	9.47	62.18

Table S3: The means of the agronomic trait of two genetic backgrounds of wheat (CS and R) under control (0 mM NaCl) and stress (250 mM NaCl) conditions

Trait	Chinese Spring lines			Roshan lines		
	**Control** (0 mM NaCl)	**Stress** (250 mM NaCl)	**Change** (%)	**Control** (0 mM NaCl)	**Stress** (250 mM NaCl)	**Change** (%)
Grain yield	1.88 [a]	0.95 [b]	-49.47	2.90 [a]	1.57 [b]	-45.86
Grain weight	2.42 [a]	1.79 [b]	-26.03	3.56 [a]	3.24 [b]	-8.99
Grains per spike	26.43 [a]	18.61 [b]	-29.59	34.48 [a]	25.87 [b]	-24.97
Spikes per plant	3.59 [a]	3.20 [b]	-10.86	2.51 [a]	2.07 [b]	-17.53
Spike length	8.07 [a]	7.49 [b]	-7.19	10.75 [a]	9.78 [b]	-9.02
Plant height	81.44 [a]	63.02 [b]	-22.62	85.37 [a]	65.61 [b]	-23.15

Trait units are shown in parentheses: grain yield (g $plant^{-1}$), grain weight (100-grain weight), grains per spike (number of grains per spike), spikes per plant (number of spikes per plant), spike length (cm), plant height (cm).

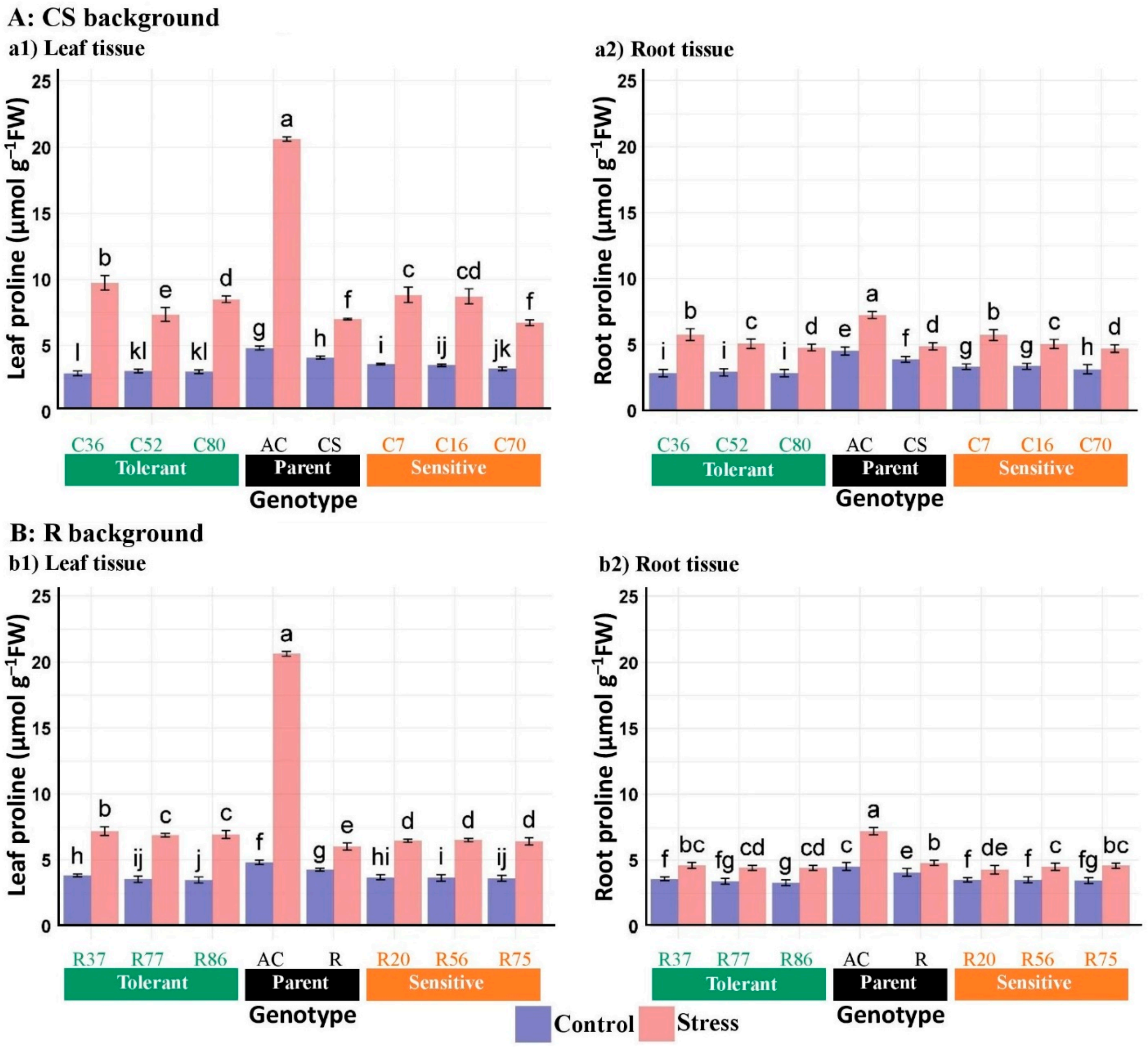

Figure S1: Mean comparison of proline content, "Chinese spring" (CS), "Roshan" (R) cultivars, *Ae. cylindrica* (AC) and their derived BC4F2 (tolerant and sensitive) lines.

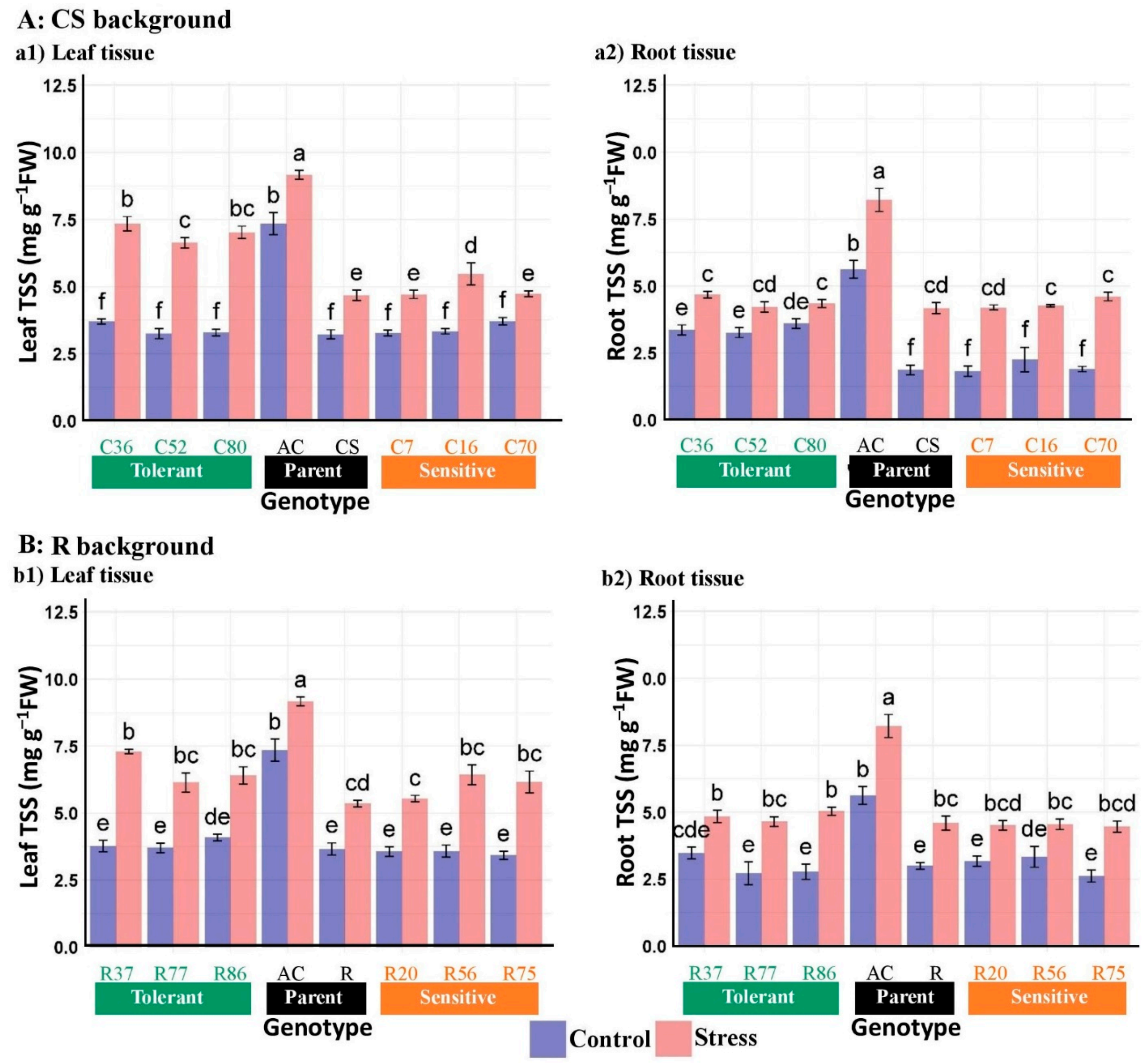

Figure S2: Mean comparison of total soluble sugars (TSS) content, “Chinese spring” (CS), “Roshan” (R) cultivars, Ae. *cylindrica* (AC) and their derived selected lines from backcrosses.

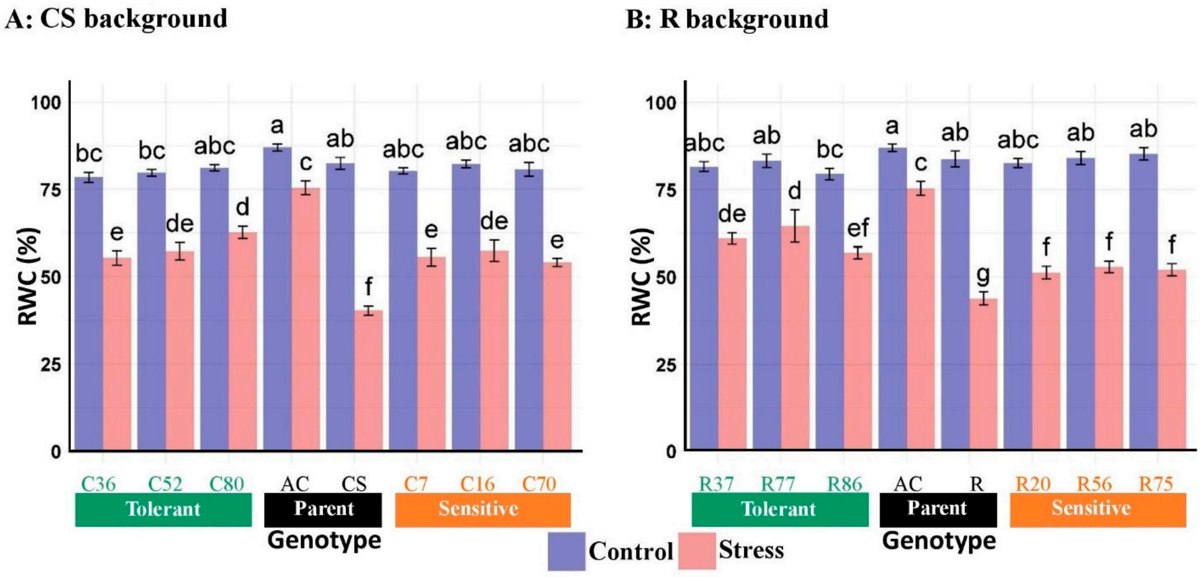

Figure S3: Mean comparison of leaf relative water content (RWC), "Chinese spring" (CS), "Roshan" (R) cultivars, Ae. *cylindrica* and their derived selected lines from backcrosses.

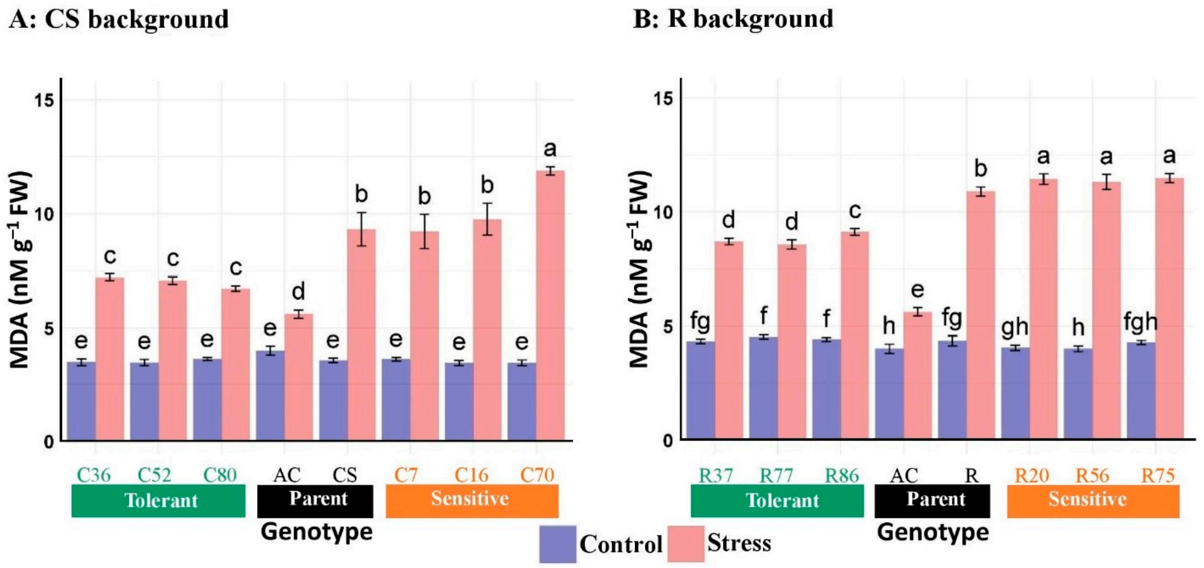

Figure S4: Mean comparison of Lipid peroxidation (MDA) content, "Chinese spring" (CS), "Roshan" (R) cultivars, Ae. *cylindrica* and their derived selected lines from backcrosses.

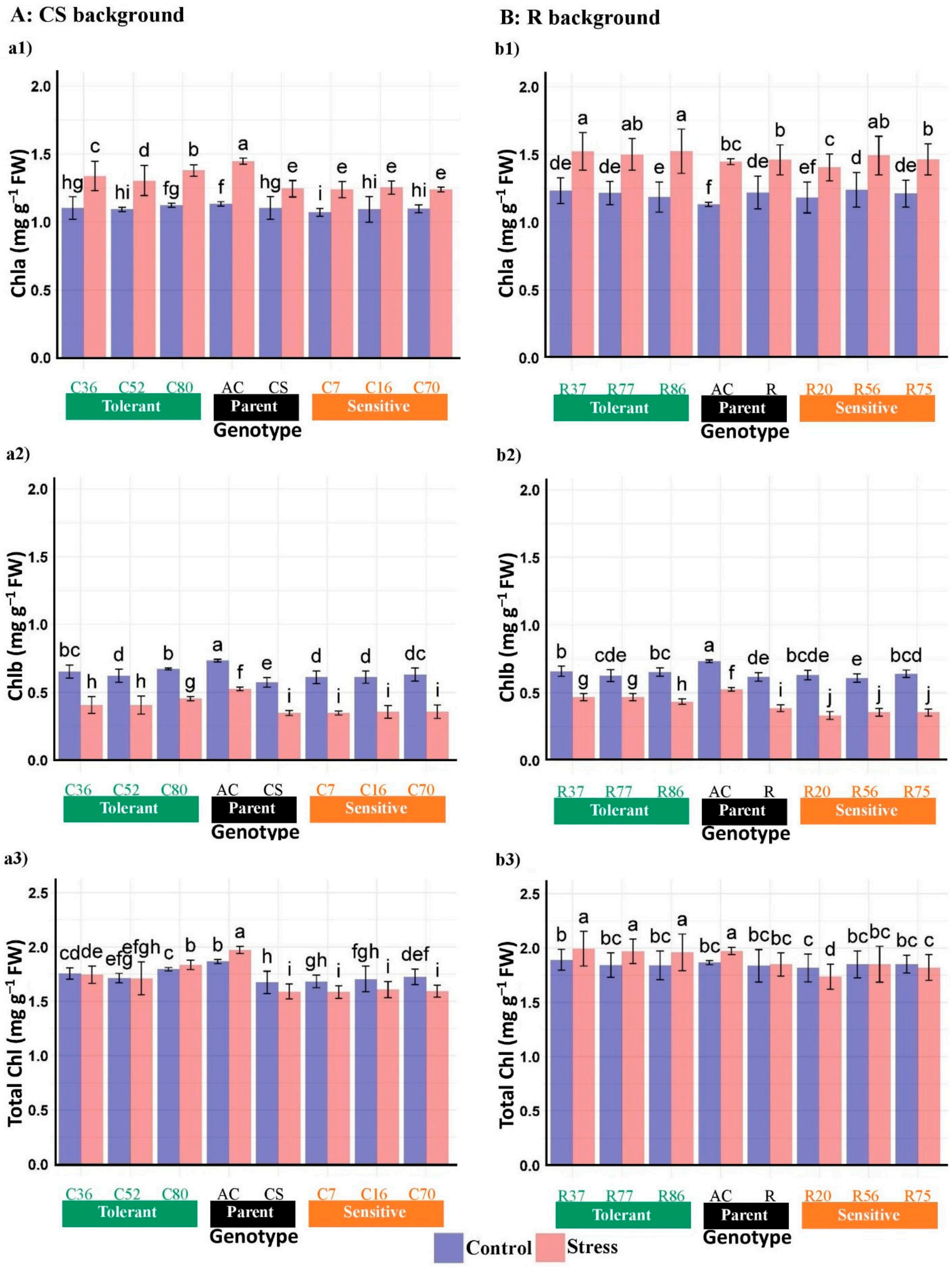

Figure S5: Mean comparison of Chlorophyll content, “Chinese spring” (CS), “Roshan” (R) cultivars, Ae. *cylindrica* and their derived selected lines from backcrosses.

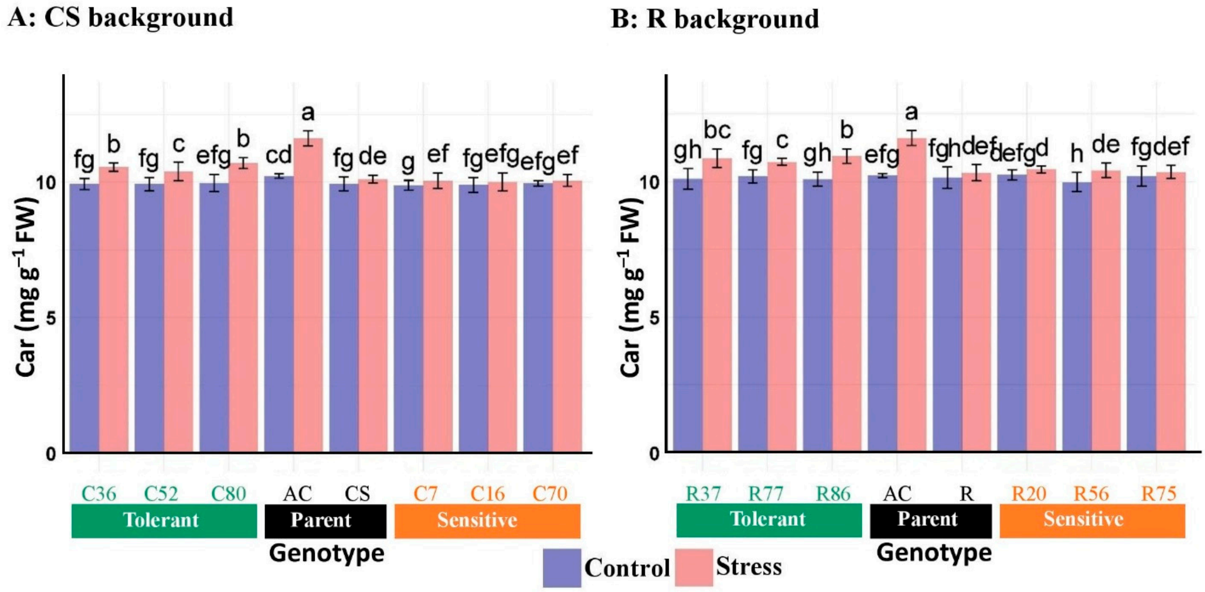

Figure S6: Mean comparison of carotenoid (Car) content, "Chinese spring" (CS), "Roshan" (R) cultivars, Ae. *cylindrica* and their derived selected lines from backcrosses.
